# Inhibiting inflammation in adipocytes accelerates mammary tumor development in mice

**DOI:** 10.1172/JCI187202

**Published:** 2025-06-17

**Authors:** Dae-Seok Kim, Toshiharu Onodera, Jan-Bernd Funcke, Kyounghee Min, Qingzhang Zhu, Qian Lin, Shiuhwei Chen, Chanmin Joung, Min Kim, R. Max Wynn, Joselin Velasco, Charlotte Lee, Megan Virostek, Chao Li, Philipp E. Scherer

**Affiliations:** 1 Touchstone Diabetes Center, University of Texas Southwestern Medical Center, Dallas, Texas, USA.; 2 Department of Adipose Management, Osaka University Graduate School of Medicine, Osaka, Japan.; 3 Barnstable Brown Diabetes and Obesity Center, University of Kentucky College of Medicine, Lexington, Kentucky, USA.; 4 Markey Cancer Center, University of Kentucky, Lexington, Kentucky, USA.; 5 Department of Biological Sciences, School of Life Sciences, Ulsan National Institute of Science and Technology, Ulsan, South Korea.; 6 Center for Hypothalamic Research, Department of Internal Medicine, University of Texas Southwestern Medical Center, Dallas, Texas, USA.

**Keywords:** Inflammation, Metabolism, Oncology, Adipose tissue

## Abstract

Proinflammatory signaling in adipocytes is essential for healthy adipose expansion, remodeling, and tissue integrity. We investigated the effects of targeting inflammation in either adipocytes or mammary gland epithelial cells, in the context of mammary tumor development, by locally expressing the antiinflammatory adenoviral RIDα/β protein complex in a cell type–specific manner. Suppression of adipocyte inflammation (RID^ad^ mice) in a mammary tumor model driven by MMTV-PyMT (PyMT-RID^ad^ mice) led to an elevated number of tumor-associated macrophages and upregulation of immunoregulatory molecules in the mammary fat pad. This was accompanied by metabolic dysfunction and abnormal mammary gland development. Importantly, this phenotype correlated with accelerated mammary tumor onset, enhanced growth, and lung metastasis. Tumors in PyMT-RID^ad^ mice exhibited upregulated CD36 expression, suggesting enhanced fatty acid uptake. Conversely, suppression of inflammation in mammary gland epithelial cells by RIDα/β expression (RID^MMTV^ mice) decelerated mammary tumor growth without affecting tumor onset or macrophage accumulation. These findings highlight the differential impact on tumor development exerted through the suppression of inflammatory signals in different cell types in the microenvironment. Our results underscore the role of the suppression of adipocyte inflammation leading to a tumor-friendly microenvironment, promoting mammary cancer progression. This study sheds light on the complex interplay between inflammation, specifically driven by the adipocyte, in breast cancer pathogenesis.

## Abstract 2

Inhibiting inflammation in tumor cells slows tumor growth, while blocking inflammation in surrounding adipocytes accelerates growth by increasing tumor-associated macrophages.

## Graphical abstract

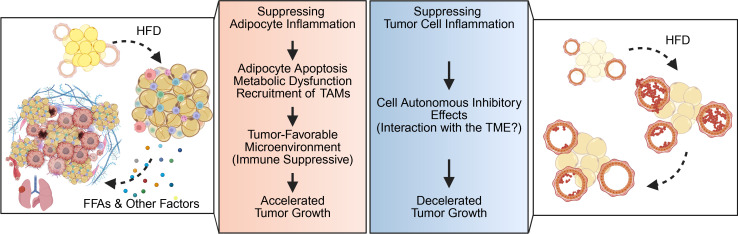

## Introduction

Our previous studies focused on the pathophysiological effects of an impaired local proinflammatory response within adipocytes, which led to increased ectopic lipid accumulation, glucose intolerance, and systemic inflammation (1). These findings came as a surprise at the time, since there was a general belief that inflammation in adipose tissue is an integral part of metabolic syndrome, and antiinflammatory actions were thought to lead to metabolic improvements. However, our findings suggested that proinflammatory signaling in the adipocyte is, in fact, required for proper adipose tissue remodeling and expansion. These findings not only deepened our overall understanding of adipose tissue dynamics in relation to inflammation, but also established an approach to effectively intervene in the inflammatory process by targeted overexpression of the adenoviral receptor internalization and degradation protein complex RIDα/β. Previous studies indicated that RIDα/β represents a transmembrane heterotrimeric complex comprising 10.4 kDa and 14.5 kDa subunits encoded by the adenovirus E3 region (2–6). This complex has been reported to robustly suppress key inflammatory pathways in vitro, including those involving LPS/TLR4, TNF-α/TNFR, and IL-1β/IL-1R (2–4). Importantly, our recent study employing adipocyte-specific doxycycline-inducible (Dox-inducible) RIDα/β transgenic mice (RID^ad^) demonstrated that suppressing inflammatory pathways in adipocytes directly affects metabolic outcomes (7). Despite a reduced weight gain, RID^ad^ mice exhibited exacerbated metabolic dysfunction, including glucose intolerance and insulin resistance, fatty liver, and reduced adiponectin levels. Consistent with previous reports (2, 3), a significant reduction in proinflammatory factors was observed; particularly notable was a reduction in M1-like macrophage markers such as *Tnf*, *Il1b*, and *Saa3*. Intriguingly, alongside this reduction, an increase in *Adgre1* expression was noted. This was accompanied by elevated levels of M2-like macrophage markers such as *Mgl1*, *Mrc1*, and *Clec10a*. These findings suggest that RIDα/β induces macrophage polarization toward an M2-like phenotype in adipose tissue through the regulation of inflammation. Numerous studies have underscored the intricate interplay between inflammatory processes and their profound impact on the initiation, progression, and metastasis of cancer (8, 9). A growing body of evidence has highlighted the important role played by chronic inflammation within the mammary environment, implicating that it creates a favorable setting for the growth and advancement of breast tumors (10–14). Furthermore, inflammatory signaling pathways have been identified as critical regulators of tumor-promoting processes, influencing aspects such as cell proliferation, angiogenesis, and immune evasion (9, 14, 15). Recent studies have explored this further, revealing details about specific molecular pathways connecting inflammation to the development of breast tumors (16, 17). Such findings highlight the crucial role of inflammatory signaling in shaping the tumor microenvironment. Despite these insights, a comprehensive examination of the tissue-specific regulation of inflammation, especially within adipocytes and tumor cells, and its implications for mammary tumor development has not been done to date in detail. Additionally, the unresolved question persists as to whether the expansion of dysfunctional adipose tissues during a high-fat diet (HFD) challenge, resulting from the suppression of inflammation in adipocytes, exerts relevant effects on the initiation and progression of mammary tumors. Our previous studies were focused on unraveling the profound connections between RIDα/β-mediated inflammation modulation and its extensive impact on metabolic regulation (1, 7).

In this study, we investigated how a selective RIDα/β-mediated suppression of inflammation in either adipocytes or mammary gland epithelial cells (i.e., the cells from which the tumor stems) affects the intricate landscape of tumorigenesis within the mammary microenvironment. To this end, we utilized RID^ad^ mice (7), in which adipocyte inflammation is suppressed, and transplanted them with mouse breast cancer cells. We also generated a transgenic model of breast cancer development in the presence of suppressed adipocyte inflammation, PyMT-RID^ad^, by combining the RID^ad^ model with mammary gland epithelial cell–specific expression of the polyomavirus middle T antigen (PyMT) (18, 19). Additionally, we created another transgenic model, PyMT-RID^MMTV^, in which both RIDα/β and PyMT were expressed specifically in mammary gland epithelial cells, allowing us to assess breast cancer development under conditions of suppressed mammary gland epithelial cell inflammation. Using these genetic mouse models, we demonstrated that suppression of adipocyte inflammation led to adipocyte apoptosis, metabolic alternation, and recruitment of tumor-associated macrophages (TAMs), collectively contributing to an immune-suppressive, tumor-promoting microenvironment. In contrast, suppression of inflammation in mammary gland epithelial cells in PyMT-RID^MMTV^ mice resulted in decelerated tumor growth without affecting tumor onset or TAM accumulation.

## Results

### RIDα/β and inflammatory gene expression in RID^ad^ mice.

**Figure 1:**
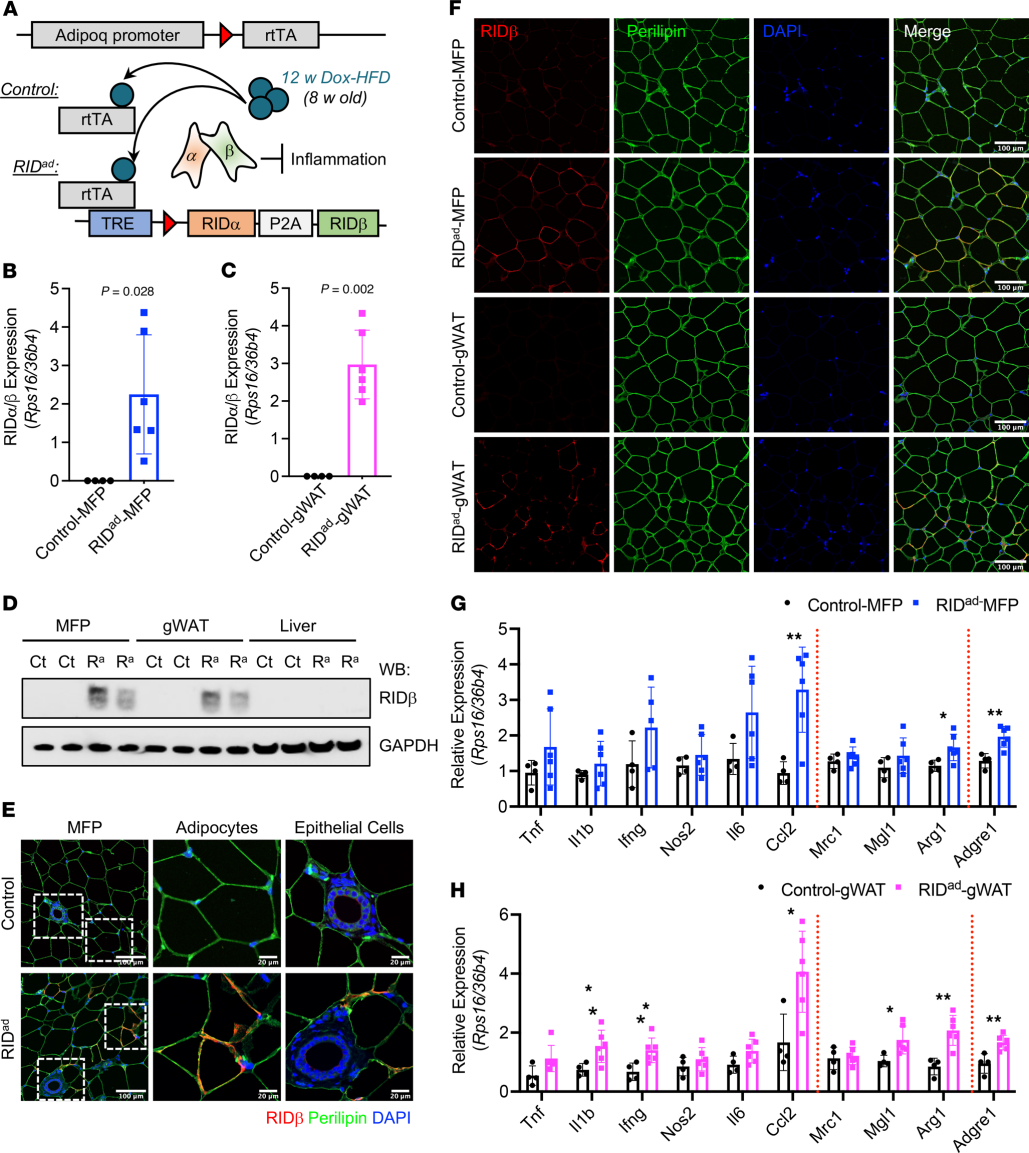
RIDα/β and inflammatory gene expression in RID^ad^ mice. (**A**) Schematic representation of adipocyte-specific, doxycycline-inducible RIDα/β transgenic mice. (**B** and **C**) qPCR analysis of RIDα/β mRNA expression in the mammary fat pad (MFP) (**B**) and gonadal white adipose tissue (gWAT) (**C**). *Rps16* and *36b4* were used for normalization. *n* = 4–6/group. (**D**) Western blot analysis of RIDβ and GAPDH protein expression in different tissues from control (Ct) and RID^ad^ (R^a^) mice. (**E**) Representative immunostaining of RIDβ and perilipin in the MFP. The magnified panels show adipocytes and mammary duct epithelial cells. Scale bar: 200 μm (left column); 20 μm (center and right columns). (**F**) Representative immunostaining of RIDβ and perilipin in the MFP and gWAT. Scale bar: 100 μm. (**G** and **H**) qPCR analysis of inflammation-related mRNA expression in the MFP (**G**) and gWAT (**H**). *Rps16* and *36b4* were used for normalization. *n* = 4–6/group. (**B**, **C**, **G**, and **H**) Data are displayed as mean ± SEM and were analyzed by unpaired 2-tailed *t* tests. *P < 0.05, **P < 0.01.

Our previous findings characterized the metabolic consequences of inflammation suppression in adipocytes using the Dox-inducible RID^ad^ mouse model (Figure 1A) (7). Here, we utilized the RID^ad^ model to investigate how modulating the inflammatory process in adipocytes contributes to breast tumor development. We first validated cell type–specific RIDα/β expression using a range of assays. Quantitative PCR (qPCR) analysis demonstrated robust RIDα/β induction in the mammary fat pad (MFP) (Figure 1B) and in the gonadal white adipose tissue (gWAT) (Figure 1C) after a 12-week challenge with a Dox-containing HFD. Western blotting confirmed the expression of RIDβ in both adipose depots (Figure 1D). Of note, although we generated distinct antibodies against RIDα and RIDβ, the RIDα antibody displayed an unacceptable level of background signal and thus was not used (data not shown). RIDβ expression was not detectable in the liver, supporting the intended restriction of transgene expression to adipocytes (Figure 1D). Immunostaining of the MFP and gWAT, particularly costaining with perilipin and RIDβ antibodies, further confirmed the adipocyte-selective induction of RIDα/β within adipose tissue (Figure 1, E and F). Importantly, RIDβ expression was not detected in mammary gland epithelial cells (Figure 1E and Supplemental Figure 1A; supplemental material available online with this article; https://doi.org/10.1172/JCI187202DS1; detailed images). In our previous study, male RID^ad^ mice exhibited a significant decrease in the expression of proinflammatory genes such as *Tnf* and *Il1b* (7). In contrast, female mice subjected to a long-term Dox-HFD regimen of 12 weeks, which is the focus of our study here, exhibited an upregulation of multiple inflammation-related genes in the MFP (Figure 1G) and gWAT (Figure 1H). However, this effect was mitigated in isolated adipocytes from the MFP (Supplemental Figure 1, B and C), suggesting that the overall induction of inflammatory genes within adipose tissue of female mice arises from other cell types, such as mammary gland epithelial cells or infiltrating immune cells, rather than from adipocytes. Notably, *Ccl2*, known for its high expression within the tumor microenvironment and its pivotal role in recruiting TAMs (20), which generally resemble M2-like macrophages, was significantly upregulated in both the MFP and gWAT (Figure 1, G and H). In line with this finding, RID^ad^ mice displayed an upregulation of M2 macrophage-related genes, including *Arg1* in both MFP and gWAT as well as *Mgl1* in gWAT. Furthermore, the pan-macrophage marker gene *Adgre1* was significantly upregulated in both the MFP and gWAT, suggesting macrophage accumulation and M2 polarization in response to prolonged suppression of adipocyte proinflammatory signaling in female mice. These results are consistent with our previous findings that macrophages in the adipose tissue of RID^ad^ mice are polarized toward an M2-like phenotype in adipose tissue (7). Collectively, these findings underscore the complex and sex-dependent nature of adipose tissue inflammation and highlight the potential impact of prolonged inflammation-modulating interventions on macrophage dynamics in females.

### Increased adipocyte apoptosis and crown-like structure formation in RID^ad^ mice.

**Figure 2:**
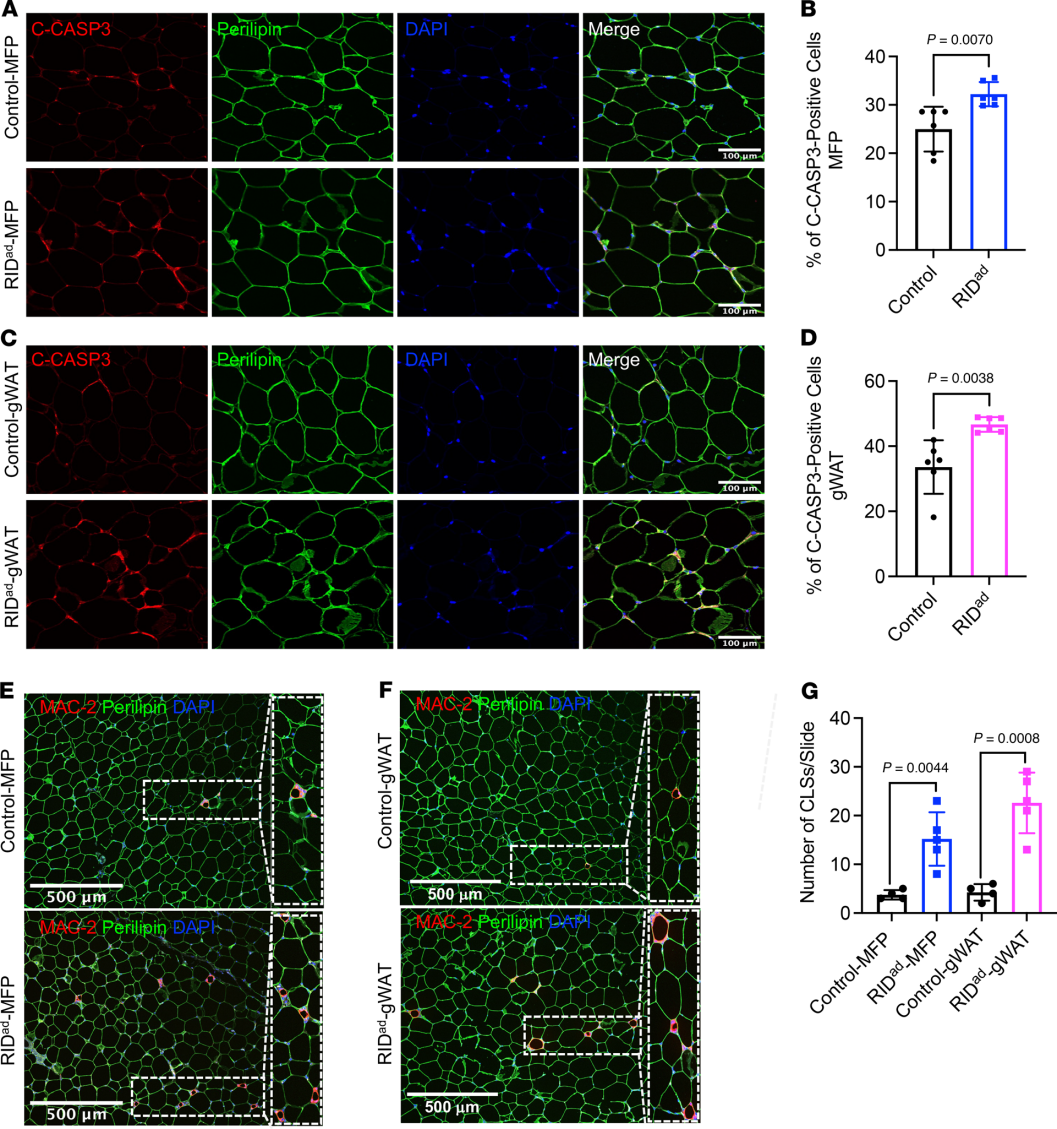
Increased adipocyte apoptosis and crown-like structure formation in RID^ad^ mice. (**A**) Representative immunostaining of cleaved caspase-3 (C-CASP3) and perilipin in the mammary fat pad (MFP). Scale bar: 100 μm. (**B**) Quantification of the percentage of cleaved C-CASP3–positive cells from the experiments shown in **A**. A total of 6 images from 3 control mice and 6 images from 3 RID^ad^ mice were quantified. (**C**) Representative immunostaining of C-CASP3 and perilipin in gonadal white adipose tissue (gWAT). Scale bar: 100 μm. (**D**) Quantification of the percentage of cleaved C-CASP3–positive cells from the experiments shown in **C**. A total of 6 images from 3 control mice and 6 images from 3 RID^ad^ mice were quantified. (**E**) Representative immunostaining of MAC-2 and perilipin in the MFP. Scale bar: 500 μm. (**F**) Representative immunostaining of MAC-2 and perilipin in gWAT. Scale bar: 500 μm. (**G**) Quantification of the number of CLSs, based on the experiments shown in **E** and **F**, was performed on 4 control mice and 5 RID^ad^ mice. Data are displayed as mean ± SEM and were analyzed by unpaired 2-tailed *t* tests (**B**, **D**, and **G**).

Macrophage-related markers were significantly upregulated in the MFP and gWAT of RID^ad^ mice that were fed Dox-HFD (Figure 1, G and H). We hypothesized that the suppression of proinflammatory signaling in adipocytes causes cell damage and unhealthy tissue expansion upon Dox-HFD challenge. This cell damage could result in increased apoptosis and subsequent recruitment of macrophages to remove cellular debris. To investigate this hypothesis, we performed immunostaining with antibodies for cleaved caspase-3 and perilipin to get a better sense of the degree of the apoptotic processes in the adipose tissues of RID^ad^ mice. Strikingly, cleaved caspase-3 was detectable at high frequency in adipocytes in the MFP (Figure 2, A and B) as well as gWAT (Figure 2, C and D) of RID^ad^ mice compared with control mice, indicating increased apoptosis. Western blotting analysis of the MFP for cleaved caspase-3 further supported this observation (Supplemental Figure 2, A and B). Although gWAT also showed increased apoptosis, the difference was not statistically significant upon quantification (Supplemental Figure 2, A and B). In adipose tissue, macrophages cluster around apoptotic adipocytes, forming crown-like structures (CLSs) (21, 22). Intriguingly, emerging evidence indicates that CLSs in adipose depots such as the MFP, particularly in the context of obesity, are associated with the development and progression of breast cancer (23). Given that we observed an increase in macrophage-related markers and adipocyte apoptosis in the MFP of RID^ad^ mice (Figure 1, G and H), we sought to determine whether apoptotic signaling is associated with exacerbated macrophage accumulation and CLS formation in MFP and gWAT. We assessed CLS formation by immunostaining using a MAC-2 antibody, which revealed an increased accumulation of CLSs in the MFP (Figure 2, E and G, and Supplemental Figure 2C; detailed images) and gWAT (Figure 2, F and G, and Supplemental Figure 2C; detailed images) of RID^ad^ mice compared with control mice. The presence of CLSs was further confirmed by H&E staining (Supplemental Figure 2D). Taken together, these observations suggest that the suppression of inflammation in MFP and gWAT during HFD challenge triggered adipocyte apoptosis, leading to the subsequent accumulation of macrophages in the MFP and gWAT of RID^ad^ mice. This dynamic interplay resulted in a fundamental change in the cellular composition within the microenvironment of the tissue.

### Increased TAM accumulation and accelerated tumor growth in the MFP of RID^ad^ mice.

**Figure 3:**
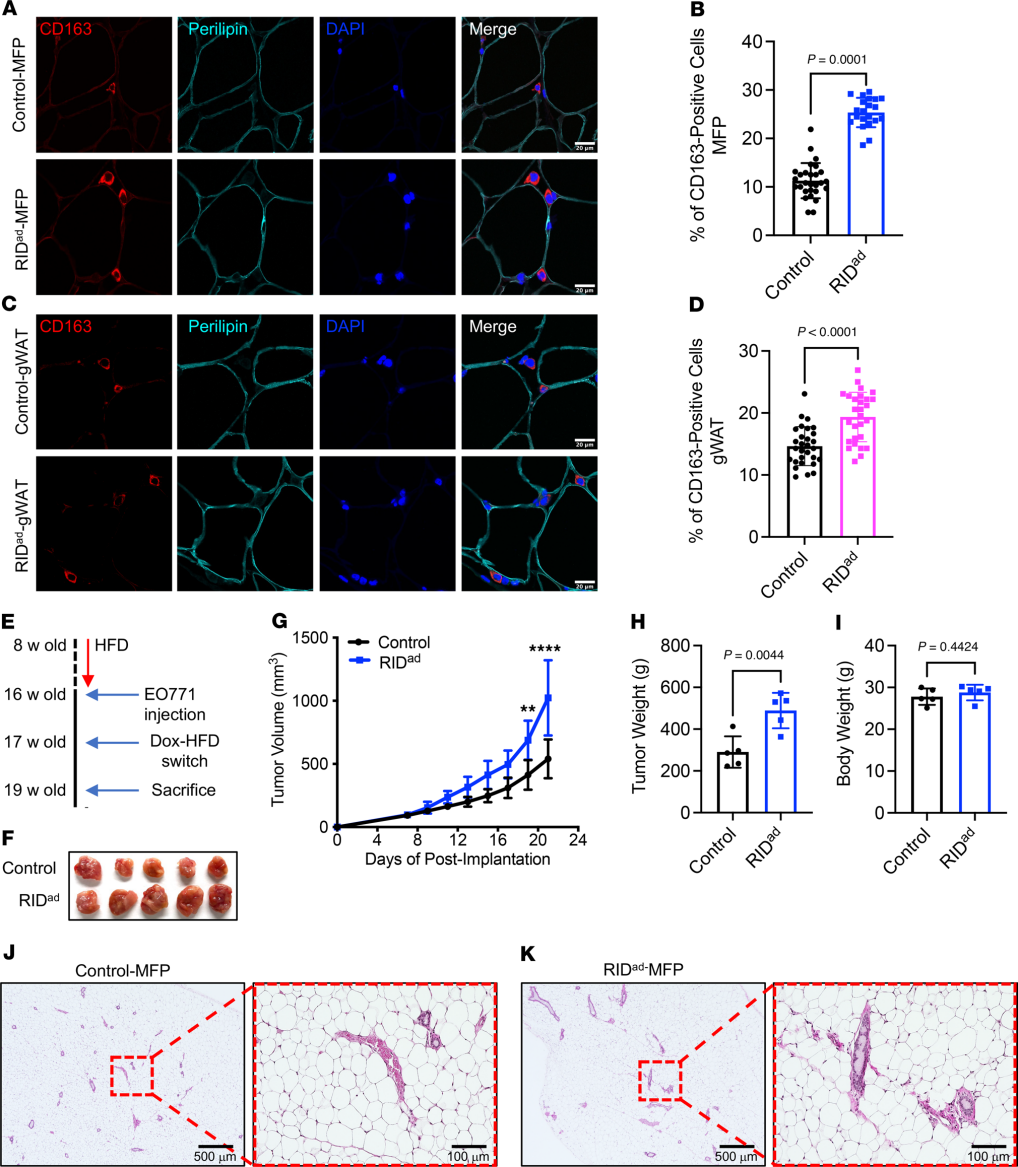
Increased TAM accumulation and accelerated tumor growth in the MFP of RID^ad^ mice. (**A**) Representative immunostaining of CD163 and perilipin in the mammary fat pad (MFP). Scale bar: 20 μm. (**B**) Quantification of the percentage of CD163-positive cells from the experiments shown in **A**. A total of 21–28 images from 3 mice per group were quantified. (**C**) Representative immunostaining of CD163 and perilipin in gonadal white adipose tissue (gWAT). Scale bar: 20 μm. (**D**) Quantification of the percentage of CD163-positive cells from the experiments shown in **C**. A total of 28–29 images from 3 mice per group were quantified. (**E**) Schematic representation of the experimental design for the EO771 syngeneic breast cancer mouse model. (**F**) Images of syngeneic tumors formed in each group. (**G**) Growth curves of transplanted EO771 cells. *n* = 5/group. (**H** and **I**) Tumor weight (**H**) and body weight (**I**) at the end of the experiment. *n* = 5/group. (**J** and **K**) H&E staining of the MFP. Scale bars: 500 μm (left); 100 μm (right). Data are displayed as mean ± SEM and were analyzed by unpaired 2-tailed *t* tests (**B**, **D**, **H**, and **I**) or 2-way ANOVA (**G**). **P = 0.01, ****P < 0.0001.

The infiltration and accumulation of TAMs within the tumor microenvironment are pivotal for tumor progression (24–26). Notably, enhanced macrophage recruitment and accumulation, along with a significant upregulation of *Ccl2*, a key factor for TAM recruitment, were observed in the MFP and gWAT of RID^ad^ mice (Figures 1 and 2). Thus, we hypothesized that the increased presence of macrophages in the MFP and gWAT of RID^ad^ mice may also reflect a greater number of TAMs, even though these mice did not carry any tumors. To assess TAM recruitment and accumulation, immunostaining with a CD163 antibody, a TAM marker (27–30), was performed. CD163 expression was significantly higher in the MFP (Figure 3, A and B) and gWAT (Figure 3, C and D) of RID^ad^ mice compared with control mice, indicative of increased TAM presence. Expression of another well-established TAM marker, CD206, was significantly higher in the MFP of RID^ad^ mice compared with control mice as well, further supporting the increased presence of TAMs (Supplemental Figure 3, A and B). To investigate the effects of suppressing adipocyte inflammation on tumor development, we employed a syngeneic breast cancer mouse model (Figure 3E). EO771 cells were injected into the MFP of both control and RID^ad^ mice that were prefed with HFD and subsequently switched to Dox-HFD for induction of RIDα/β expression. Notably, suppression of adipocyte inflammation significantly enhanced the growth of syngeneic tumors in RID^ad^ mice compared with control mice (Figure 3, F–H), without affecting body weight (Figure 3I) or the composition or gross morphology of the MFP (Figure 3, J and K). In line with this observation, conditioned medium (CM) collected from in vitro–differentiated adipocytes of RID^ad^ mice promoted cancer cell proliferation and migration in several human breast cancer cell lines (Supplemental Figure 4, A–C). Interestingly, CM from in vitro–differentiated adipocytes of RID^ad^ mice (C57BL/6 mice background) induced the proliferation and migration of EO771 cells (C57BL/6 mice derived) but not MET1 cells (FVB mice derived), suggesting a background-specific effect of CM (Supplemental Figure 4, D–F).

### Earlier mammary tumor onset, accelerated tumor growth, and a higher incidence of lung metastasis in PyMT-RID^ad^ mice.

**Figure 4:**
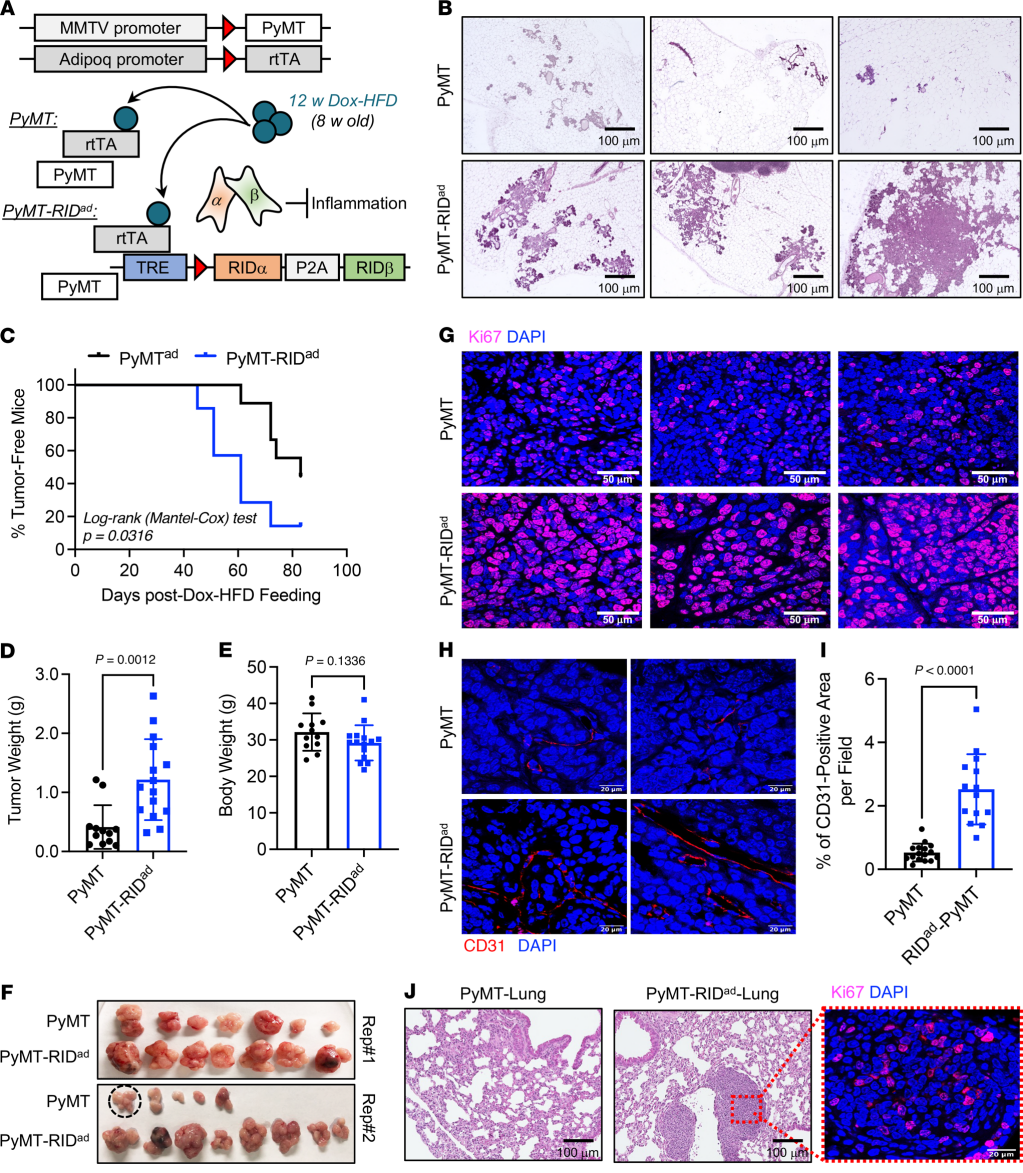
Earlier mammary tumor onset, accelerated tumor growth, and a higher incidence of lung metastasis in PyMT-RID^ad^ mice. (**A**) Schematic representation of the generation of PyMT-RID^ad^ mice by introducing RID^ad^ mice into the MMTV-PyMT mammary tumor model. (**B**) Analysis of the mammary fat pad (MFP) in PyMT-RID^ad^ mice revealed abnormalities in mammary gland development, as evidenced by H&E staining. Scale bars: 100 μm. Representative samples are shown. (**C**) Kaplan-Meier tumor-free mouse curves from 2 independent experiments. *n* = 13–16/group. (**D** and **E**) Tumor weight (**D**) and body weight (**E**) at the end of the experiment. *n* = 13–16/group. (**F**) Images of tumors formed in each group at the end of the experiments (mice approximately 20 weeks old). A circle indicates multiple tumors that were harvested from a single mouse. (**G**) Representative immunostaining of Ki67 in the tumors. *n* = 3/group. Scale bars: 50 μm. (**H**) Representative immunostaining of CD31 in the tumors. Scale bars: 50 μm. (**I**) Quantification of the percentage of CD163-positive area from the experiments shown in **H**. A total of 14–16 images from 3 mice per group were quantified. (**J**) H&E staining shows metastatic breast tumor development in the lungs of PyMT-RID^ad^ mice. A magnified image of Ki67-immunostained tumor is provided. Scale bars: 100 μm. Data are displayed as mean ± SEM and were analyzed by unpaired 2-tailed *t* tests (**D**, **E**, and **I**).

To study an autochthonous model of breast cancer development, we generated PyMT-RID^ad^ mice, which are RID^ad^ mice carrying the MMTV-PyMT transgene (Figure 4A). Female MMTV-PyMT spontaneously develop breast cancer, providing an ideal model for exploring the detailed outcomes of suppressing adipocyte inflammation (18). Our analysis focused on the mammary fat pad (MFP) rather than gonadal white adipose tissue (gWAT) because breast tumors predominantly originate and develop in the MFP. First, examination of the MFP in PyMT-RID^ad^ mice fed Dox-HFD revealed abnormal mammary gland development compared with PyMT mice, as determined by H&E staining (Figure 4B and Supplemental Figure 5A; magnified images). Second, PyMT-RID^ad^ mice showed a significantly earlier mammary tumor onset, developing palpable tumors at a younger age than PyMT mice (Figure 4C). PyMT-RID^ad^ mice exhibited significantly faster tumor growth compared with PyMT mice (Figure 4D) without any effect on body weight (Figure 4E). Tumors isolated from each group confirmed that RIDα/β overexpression in adipocytes promoted tumor growth (Figure 4F). Additionally, tumors from PyMT-RID^ad^ mice displayed a notable increase in actively proliferating Ki67-positive tumor cells (Figure 4G and Supplemental Figure 5, B and C). Also, the development of blood vessels within the tumor lesions compared with PyMT mice was enhanced, as indicated by CD31 staining and H&E staining (Figure 4, H and I, and Supplemental Figure 5D). These findings suggest that the suppression of inflammation in adipocytes stimulates both tumor development and growth. Interestingly, the number of macrophages at this late stage of tumor development was significantly decreased in the MFP of PyMT-RID^ad^ mice compared with PyMT mice, which contrasts with our observations in RID^ad^ mice (Supplemental Figure 5, E–G). Increased nutritional competition between tumor cells and macrophages has been reported to limit macrophage survival and function in the tumor microenvironment (31–33). In PyMT-RID^ad^ mice, where the MFP exhibits extensive tumor development, such competition may account for the reduced macrophage presence observed under this condition. Additionally, tumors from PyMT-RID^ad^ mice expressed levels of estrogen receptor α (ERα) comparable to PyMT mice, indicating that the rapid tumor development and progression in PyMT-RID^ad^ mice occurs in an ERα-independent manner (Supplemental Figure 5H). Finally, examination of the lungs of PyMT-RID^ad^ mice revealed metastatic tumor development, a phenomenon not detectable in PyMT mice at this time point (Figure 4J). In conclusion, the suppression of inflammation in adipocytes in PyMT-RID^ad^ mice promoted an earlier mammary tumor onset, accelerated tumor growth, and fostered the formation lung metastases.

### Metabolic dysfunction and increased lipid uptake in tumors of PyMT-RID^ad^ mice.

**Figure 5:**
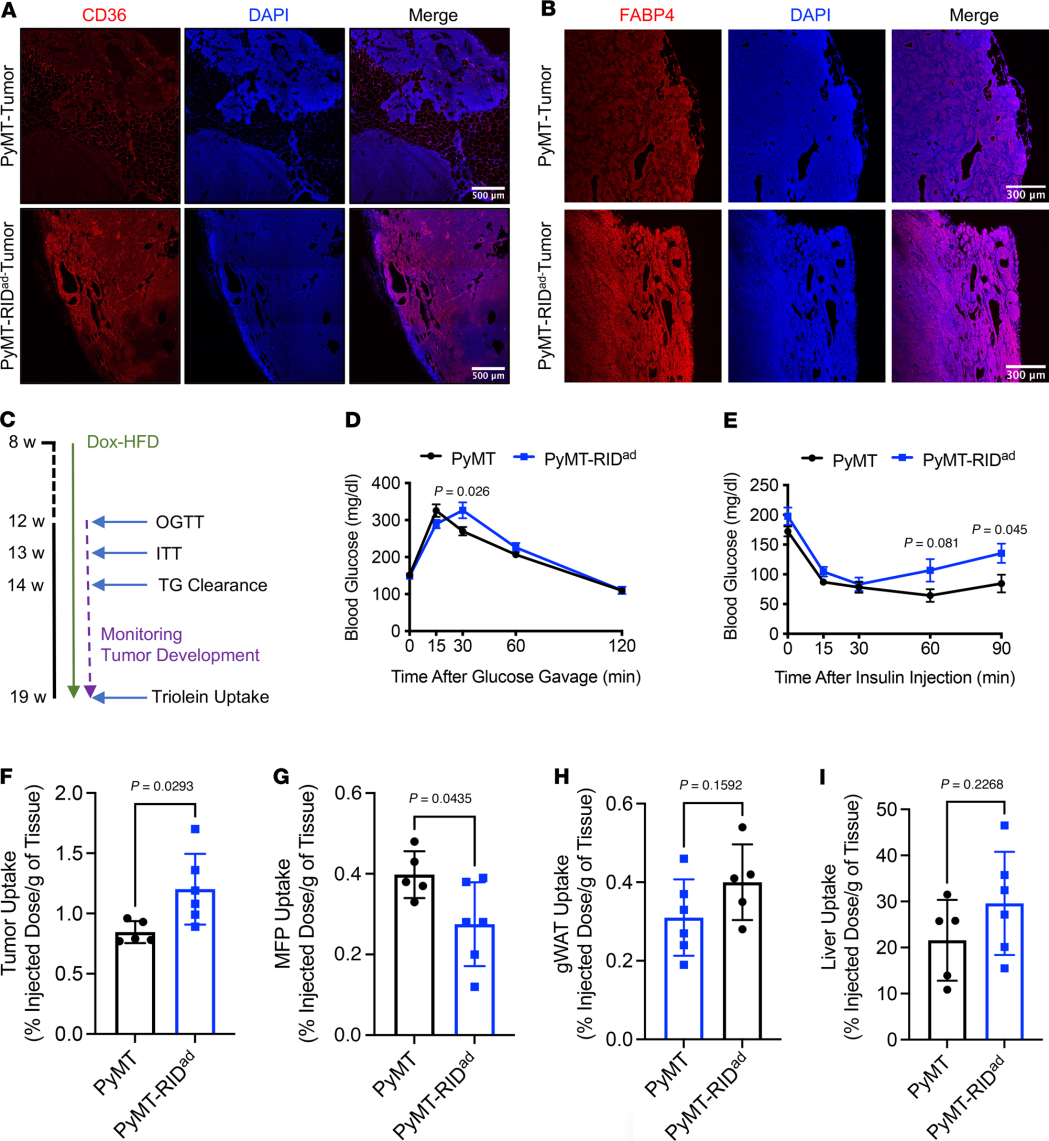
Metabolic dysfunction and increased lipid uptake in tumors of PyMT-RID^ad^ mice. (**A**) Representative immunostaining of CD36 in the tumors. *n* = 3/group. Scale bars: 500 μm. The image of a different area of the same tissue section is shown in Supplemental Figure 7. (**B**) Representative immunostaining of FABP4 in the tumors. *n* = 3/group. Scale bars: 300 μm. (**C**) Schematic representation of the experimental design for the metabolic phenotyping, tumor development, and triolein uptake. *n* = 5–6/group. (**D**) Oral glucose tolerance test. (**E**) Intraperitoneal insulin tolerance test (ITT). (**F**–**I**) Triolein uptake into the tumor (**F**), mammary fat pad (MFP; **G**), gonadal white adipose tissue (gWAT; **H**), and liver (**I**). *n* = 5–6/group. Data are displayed as mean ± SEM and were analyzed by unpaired 2-tailed *t* tests (**D**–**I**).

We performed RNA-Seq to further elucidate the changes in tumor gene expression occurring in the context of suppressed adipocyte inflammation (Supplemental Figure 6A). Among the top 5 pathways identified in the RNA-Seq analysis, the AMPK signaling pathway, known for negatively regulating the mTOR pathway and thereby inhibiting cancer cell survival and proliferation, was significantly downregulated (Supplemental Figure 6B; top) (34). Although not among the top 5, tumors from PyMT-RID^ad^ mice displayed a significant upregulation of Wnt and PI3K-Akt signaling pathways (Supplemental Figure 6B; bottom), which are critical regulators of tumor progression (35). Our RNA-Seq findings thus partially explain the heightened proliferation and more aggressive tumor phenotype observed in PyMT-RID^ad^ mice. Given that highly proliferative tumors require increased fatty acid uptake to support their growth, we next hypothesized that genes involved in fatty acid metabolism would be upregulated in tumors from PyMT-RID^ad^ mice. To test this hypothesis, we used immunostaining to examine the protein expression of key factors involved in fatty acid uptake and metabolism, including CD36 and FABP4, in tumor tissues. We found that CD36 was highly expressed in tumors from PyMT-RID^ad^ mice compared with PyMT mice (Figure 5A and Supplemental Figure 7; image of a different area of the same tissue section). FABP4 displayed a slightly higher expression in PyMT-RID^ad^ tumors as well (Figure 5B). CD36 plays a critical role in lipid metabolism by facilitating the uptake of free fatty acids. These results suggest that the tumors in PyMT-RID^ad^ mice may rely on fatty acids to support their growth. To test whether interference with CD36 blocks tumor cell proliferation in PyMT-RID^ad^ mice, we isolated tumor cells from PyMT and PyMT-RID^ad^ mice and cultured them in vitro (Supplemental Figure 8A). The tumor cells formed colonies at passage 0 in both groups, and after passaging, tumor cells from PyMT mice exhibited poor colony formation, whereas those from PyMT-RID^ad^ mice appeared to form colonies well (Supplemental Figure 8B, passage 2). Therefore, we only performed further experiments using tumor cells from PyMT-RID^ad^ mice. Treatment with the CD36 inhibitor sulfosuccinimidyl oleate (SSO) slightly reduced tumor cell proliferation when used at higher concentrations, indicating that tumor cells from PyMT-RID^ad^ mice appeared to rely on CD36 expression on their surface (Supplemental Figure 8C). To further elucidate changes in systemic and local metabolism in PyMT-RID^ad^ mice, we conducted a comprehensive evaluation of metabolic parameters, including systemic glucose tolerance, insulin sensitivity, lipid clearance, and triolein uptake, while monitoring tumor development (Figure 5C). We found that PyMT-RID^ad^ mice exhibited systemic metabolic dysfunction, including mild glucose intolerance (Figure 5D) and moderate insulin resistance (Figure 5E) compared with PyMT mice. To assess lipid clearance in these animals, we performed oral triglyceride challenges (Supplemental Figure 9). The fasting plasma and free fatty acid levels were comparable between PyMT and PyMT-RID^ad^ mice, suggesting that systemic lipid metabolism was not altered. However, a triolein uptake experiment revealed that the tumors took up a significantly higher amount of triolein (Figure 5F), while the MFP itself displayed a lower triolein uptake in PyMT-RID^ad^ mice (Figure 5G). These observations suggest that metabolically dysfunctional adipocytes in the MFP of PyMT-RID^ad^ mice had a reduced ability to take up and metabolize lipids, whereas the tumor lesions showed a greater ability to do so. This aligns well with our observations of the highly proliferative character of the tumors in PyMT-RID^ad^ mice (Figure 4 and Supplemental Figure 10), as shown by their high expression of CD36 (Figure 5A). Other tissues, including gWAT (Figure 5H) and the liver (Figure 5I), did not show significant changes in triolein uptake, corroborating the functional and direct interaction between the mammary adipocytes and tumor cells. Taken together, our data indicate that the metabolically dysfunctional mammary environment in PyMT-RID^ad^ mice promoted the development of highly proliferative tumors that showed an elevated capacity for fatty acid uptake.

### Increased CLS formation and TAM accumulation at early stages of tumor development in PyMT-RID^ad^ mice.

**Figure 6:**
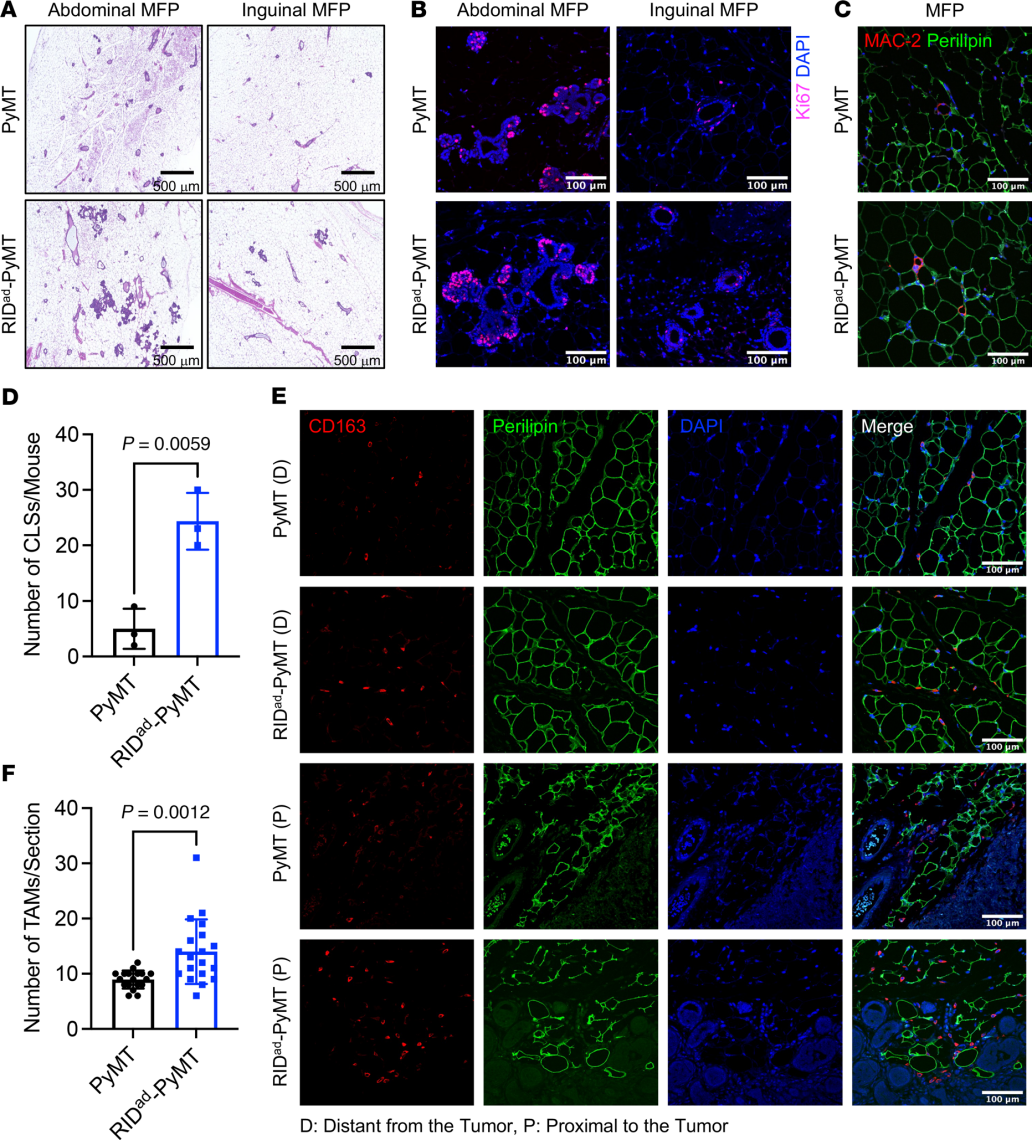
Increased CLS formation and TAM accumulation at early stages of tumor development in PyMT-RID^ad^ mice. (**A**) H&E staining of the mammary fat pad (MFP), encompassing both abdominal and inguinal pads. *n* = 3/group. Scale bars: 500 μm. (**B**) Representative immunostaining of Ki67 in the MFP. *n* = 3/group. Scale bars: 100 μm. Detailed images are shown in Supplemental Figure 11. (**C**) Representative immunostaining of MAC-2 and perilipin in the MFP. Scale bars: 100 μm. (**D**) Quantification of the number of crown-like structures (CLSs) from the experiments shown in **C**. *n* = 3/group. (**E**) Representative immunostaining of CD163 and perilipin in the MFP. Regions distant from and proximal to the tumor are shown. Scale bars: 100 μm. (**F**) Quantification of the number of tumor-associated macrophages (TAMs) from the experiments shown in **E**. *n* = 3/group. Data are displayed as mean ± SEM and were analyzed by unpaired 2-tailed *t* tests (**D** and **F**).

After 12 weeks of Dox-HFD feeding, the MFP in PyMT-RID^ad^ mice displayed a notable proliferation of mammary gland tissue and the emergence of tumors (Figure 4B). This proliferation significantly (p<0.0001) altered the accumulation of macrophages within the MFP (Supplemental Figure 5, E and F). Furthermore, the widespread presence of actively proliferating mammary gland components and tumor cells within the MFP posed challenges for obtaining pure adipose samples suitable for RNA-Seq. Therefore, we investigated the early-stage tumor-associated pathological changes in PyMT-RID^ad^ mice after a short-term Dox-HFD challenge of only 4 weeks to identify potential factors that could stimulate rapid tumor growth while avoiding the confounding effects of tumor contamination observed at the 12-week stage. First, we observed that the abdominal MFP in PyMT-RID^ad^ mice already showed slightly proliferative mammary gland epithelial cells and early tumor development compared with PyMT mice (Figure 6A, left), while the inguinal MFP showed barely detectable mammary gland proliferation and tumor development (Figure 6A, right). Additionally, immunostaining for Ki67 revealed a moderate number of proliferating tumor cells in the abdominal MFP, whereas only a small number were detected in the inguinal MFP, indicating markedly lower tumor cell contamination in the inguinal MFP (Figure 6B and Supplemental Figure 11; detailed images). These observations suggest that a short-term HFD challenge of 4 weeks serves as an effective model for investigating early dynamic changes in the MFP preceding the onset of highly proliferative mammary glands and mammary tumor development. To this end, we collected one side of the entire MFP for analysis of CLS formation, TAM recruitment, and expression of cleaved caspase-3. To assess CLS formation, we conducted MAC-2 immunostaining, which revealed an increased abundance of MAC-2–positive cells forming CLSs in the MFP of PyMT-RID^ad^ mice compared with PyMT mice (Figure 6, C and D, and Supplemental Figure 12; detailed images). Additionally, to assess TAM recruitment and accumulation, CD163 and CD206 immunostaining were carried out. Expression of both CD163 (Figure 6, E and F) and CD206 (Supplemental Figure 13) were significantly higher in the MFP of PyMT-RID^ad^ mice compared with PyMT mice. Interestingly, CD163-positive cells were highly enriched in the proximal region from the tumor compared with distal regions, indicative of an increased number of TAMs more closely located near the tumor lesions (Figure 6E). Importantly, we observed recruitment of TAMs in the MFPs of both groups of mice, that is, with and without tumor lesions (RID^ad^ in Figure 3A and PyMT-RID^ad^ in Figure 6E), reflecting an overall tumor-favorable environment and suggesting that TAM recruitment may be a critical factor in stimulating tumor growth in our mouse model. However, we did not observe an enhanced induction of apoptosis at this earlier stage, as indicated by the lack of signal for cleaved caspase-3 in PyMT-RID^ad^ mice (data not shown). Macrophages within adipose tissue aggregate to form CLS around adipocytes undergoing apoptosis (22). Despite the absence of cleaved caspase-3, we observed a significant increase in CLSs during the short-term HFD challenge in PyMT-RID^ad^ mice, suggesting that adipocytes in these mice may undergo apoptosis at higher rates, resulting in an increased recruitment of macrophages (Figure 6, C and D).

### Inflammation suppression in adipocytes alters the tumor microenvironment, metabolism, and immune landscape in PyMT-RID^ad^ mice.

**Figure 7:**
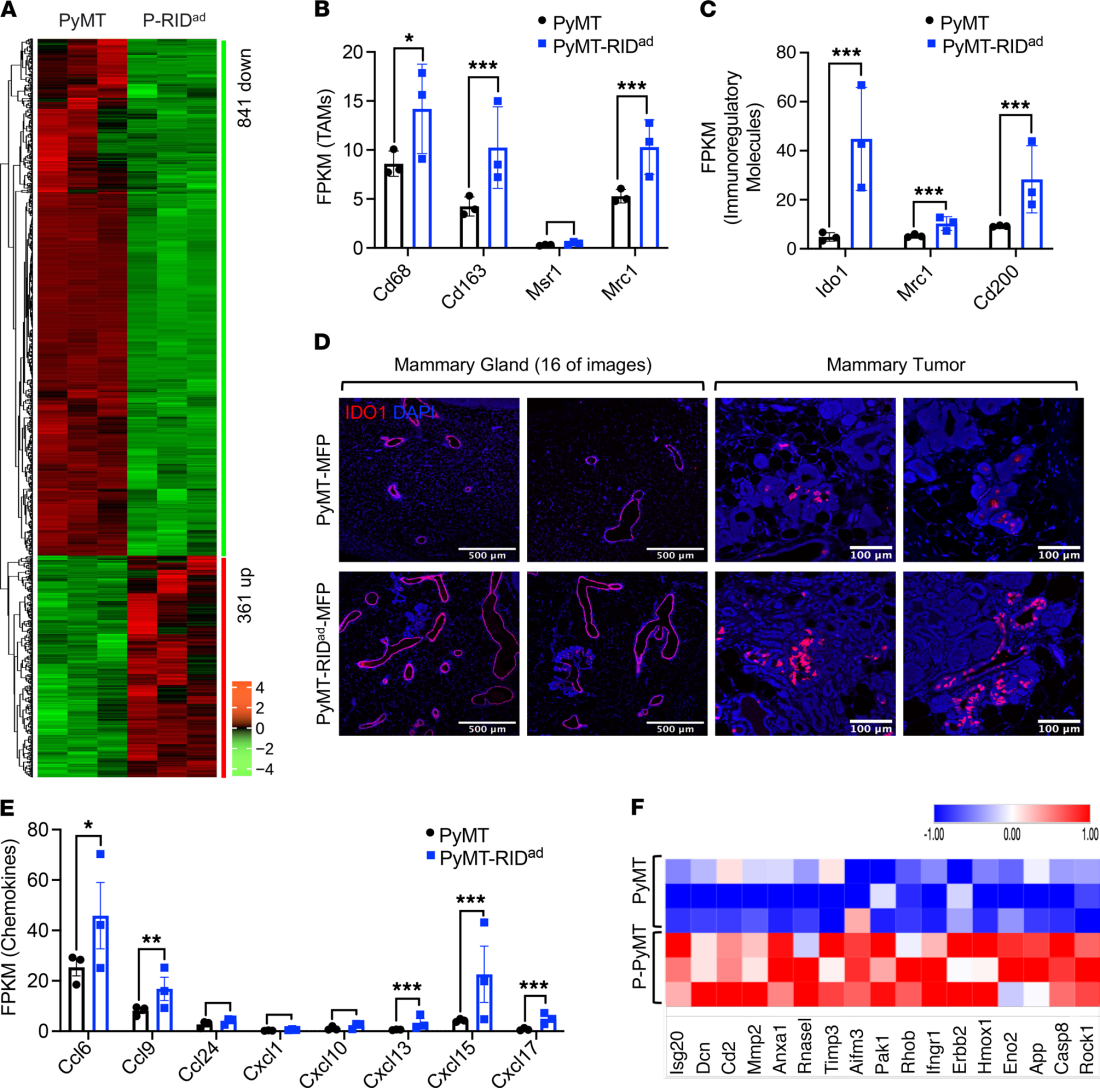
Inflammation suppression in adipocytes alters the tumor microenvironment, metabolism, and immune landscape in PyMT-RID^ad^ mice. (**A**) Heatmap of differentially expressed genes by RNA-Seq in the mammary fat pad (MFP). *n* = 3/group. (**B**) RNA-Seq analysis of tumor-associated macrophage (TAM) markers. (**C**) RNA-Seq analysis of immunoregulatory molecules. (**D**) Representative immunostaining of IDO1 in the MFP, specifically in mammary gland and tumor regions. *n* = 2–3/group. Scale bars: 500 μm (mammary gland); 300 μm (mammary tumor left); 100 μm (mammary tumor right). Detailed images are shown in Supplemental Figure 15. (**E**) RNA-Seq of chemoattractant molecules. (**F**) Heatmap of differentially expressed apoptosis-related genes by RNA-Seq in the MFP. Data are displayed as mean ± SEM and were analyzed by unpaired 2 tailed *t* tests (**B**, **C**, and **E**). *P < 0.05, ***P < 0.001.

To understand how early alterations of the MFP in PyMT-RID^ad^ mice contribute to the acceleration of mammary tumor development and progression, we performed RNA-Seq on the MFP of PyMT-RID^ad^ and PyMT mice fed Dox-HFD for only 4 weeks (Supplemental Figure 14). Given the relatively lower contamination of the inguinal MFP with tumor cells, we utilized that portion of the MFP for our analysis (Figure 6, A and B, and Supplemental Figure 14). RNA-Seq revealed that the suppression of inflammation in adipocytes altered the expression of hundreds of genes in PyMT-RID^ad^ mice compared with PyMT mice (Figure 7A and Supplemental Figure 14). Initially, we observed that multiple TAM-related genes, including *Cd68*, *Cd163*, and *Mrc1*, but not *Msr1*, were highly upregulated in PyMT-RID^ad^ mice (Figure 7B). This finding aligns well with our histological observations (Figure 6E) and highlights the recruitment and accumulation of TAMs in the MFP of PyMT-RID^ad^ mice. This phenomenon represents one of the most prominent consequences of inflammation suppression in adipocytes in our model. Importantly, the MFP of PyMT-RID^ad^ mice also exhibited an enrichment of immunoregulatory molecules such as *Ido1*, *Mrc1*, and *Cd200*, suggesting a shift toward a generally immunosuppressive tumor microenvironment (Figure 7C). The upregulation of *Mrc1* implies an increase in protumorigenic M2 macrophages (36), and elevated *Cd200* expression may contribute to immune evasion by inhibiting antitumor immune responses (37). *Ido1* in turn is known to facilitate tumor immune escape by suppressing T cell activity and promoting Treg expansion (38–40). Immunostaining confirmed substantially higher IDO1 expression in both mammary gland epithelial cells and tumor cells in PyMT-RID^ad^ mice compared with PyMT controls (Figure 7D and Supplemental Figure 15; detailed images). Despite these immunosuppressive changes, we did not observe any detectable differences in M1 macrophages, DCs, NK cells, or T cells in the MFP of RID^ad^ mice (Supplemental Figure 16) and PyMT-RID^ad^ mice (Supplemental Figure 17) when compared with their respective controls. These findings suggest that RID^ad^ promotes an immunosuppressive tumor microenvironment through an upregulation of IDO1 in yet to be identified cell types as well as an augmented recruitment of immunomodulatory TAMs, rather than through direct alterations in other immune cell populations. We indeed observed an upregulation of multiple chemokines in the MFP of PyMT-RID^ad^ mice that may facilitate macrophage recruitment (Figure 7E). Apoptosis-related genes were also highly enriched in the MFP of PyMT-RID^ad^ mice (Figure 7F and Supplemental Figure 18), supporting our hypothesis that inflammation suppression by RIDα/β overexpression induces increased adipocyte apoptosis. Furthermore, genes related to mammary gland development, mammary duct morphogenesis, tumor progression, and metastasis were significantly upregulated in the MFP of PyMT-RID^ad^ mice, consistent with histological evidence of highly proliferative mammary glands (Supplemental Figure 19, A and B). In contrast, many genes involved in glycolysis, glycogen metabolism, and browning were downregulated after inflammation suppression in adipocytes (Supplemental Figure 19, A and B). This reduction in glycolytic activity may increase nutrient availability within the tumor microenvironment, thereby facilitating tumorigenesis. Interestingly, *Il12a*, a critical regulator of inflammation (41), was significantly downregulated in the MFP of PyMT-RID^ad^ mice (Supplemental Figure 19C). This suggests that *Il12a* may be one of the main targets affected by RIDα/β overexpression, leading to the suppression of inflammation. Subsequent pathway analysis (Supplemental Figure 20) revealed impaired insulin signaling in the MFP of PyMT-RID^ad^ mice marked by downregulation of the PI3K-Akt pathway (Supplemental Figure 20C), further linking RIDα/β overexpression to insulin resistance. Intriguingly, RID^ad^ mice without PyMT subjected to 12 weeks of Dox-HFD feeding showed increased adipocyte size (Supplemental Figure 21A). Similarly, at 4 weeks in PyMT-RID^ad^ mice, prior to tumor development, adipocyte hypertrophy was evident as well (Supplemental Figure 21B). This suggests that adipocyte enlargement may be at the root of diminished insulin signaling in the MFP of PyMT-RID^ad^ mice (42). These findings suggest that impaired insulin signaling and metabolic dysfunction precede and promote tumor development. Interestingly, at 12 weeks, when PyMT-RID^ad^ mice exhibited a tumor burden, overall adipocyte size remained unchanged when randomly assessed (Supplemental Figure 21C). However, adipocytes adjacent to tumors appeared smaller, likely due to increased lipolysis and metabolic demand within the tumor microenvironment (Supplemental Figure 21D). These opposing effects, RIDα/β-induced hypertrophy versus tumor-driven adipocyte reduction, may offset one another, explaining the lack of a significant difference in overall adipocyte size. Collectively, these findings demonstrate that suppression of inflammation in adipocytes profoundly reprograms the metabolic and immune landscape of the MFP, promoting an immunosuppressive tissue microenvironment that benefits tumor development and growth.

### Decelerated mammary tumor growth but unchanged tumor onset and tissue macrophage accumulation in PyMT-RID^MMTV^ mice.

**Figure 8:**
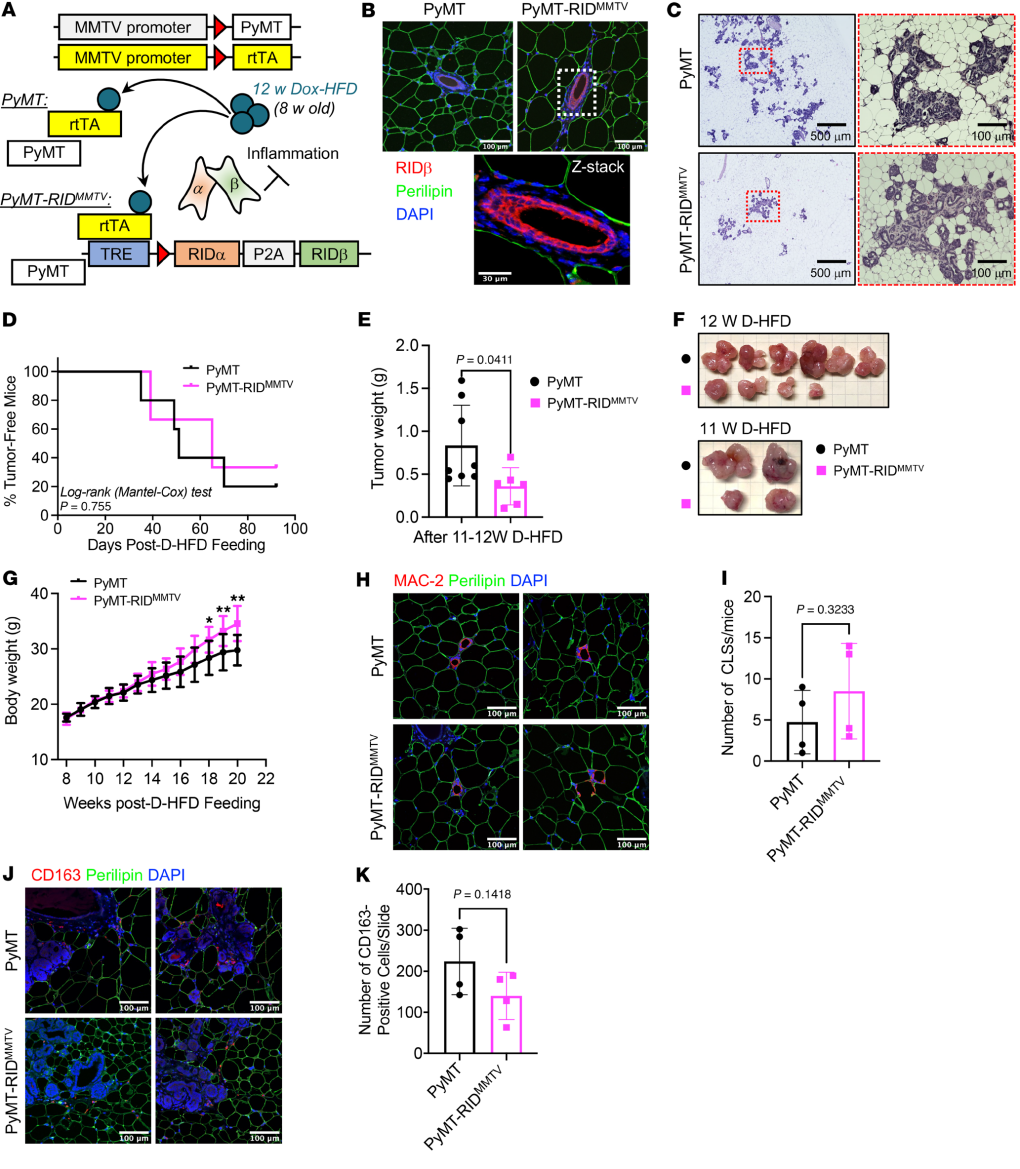
Decelerated mammary tumor growth but unchanged tumor onset and tissue macrophage accumulation in PyMT-RID^MMTV^ mice. (**A**) Schematic representation of the generation of PyMT-RID^MMTV^ mice by introducing mammary gland epithelial cell–specific, doxycycline-inducible RIDα/β transgenic mice into the MMTV-PyMT mammary tumor model. *n* = 7–9/group. (**B**) Representative immunostaining of RIDβ and perilipin in the mammary fat pad (MFP). Scale bars: 100 μm (top); 50 μm (bottom). (**C**) PyMT-RID^MMTV^ mice reveal reduced abnormalities in mammary gland development, as evidenced by H&E staining. Scale bars: 500 μm (left); 100 μm (right). (**D**) Kaplan-Meier tumor-free mouse curves from 2 independent experiments. *n* = 7–9/group. (**E**) Tumor weight at the end of the experiment. *n* = 6–8/group. (**F**) Images of tumors formed in each group at the end of the experiments (mice approximately 19–20 weeks old). In each group, 2 mice had to be euthanized a week earlier because of a high tumor burden. *n* = 6–8/group. (**G**) Body weight. *n* = 7–9/group. Two-way ANOVA. *P = 0.0, **P < 0.01. (**H**) Representative immunostaining of MAC-2 and perilipin in the MFP. Scale bars: 100 μm. (**I**) Quantification of the number of crown-like structures (CLSs) from the experiments shown in **H**. *n* = 4/group. (**J**) Representative immunostaining of CD163 and perilipin in the MFP. Scale bars: 100 μm (**K**) Quantification of the number of tumor-associated macrophages (TAMs) from the experiments shown in **J**. *n* = 4/group. Data are displayed as mean ± SEM and were analyzed by unpaired 2-tailed *t* tests (**E**, **G**, **I**, and **K**).

Proinflammatory factors, such as TNF-α, IL-1β, and IL-6, influence the development and progression of mammary tumors (8, 14). Chronic inflammation has been linked to increased breast cancer development, contributing to the processes of initiation, promotion, and metastasis of tumors (8, 14). We observed that the suppression of inflammation in adipocytes significantly increased breast tumor onset, growth, and metastasis (Figure 4). These observations indicate that the inhibition of inflammation in different tissues may have distinct effects on breast tumor development and progression. To explore the specific tumor-associated pathological outcomes of suppression of inflammation in mammary gland epithelial cells, we generated mammary gland epithelial cell–specific, Dox-inducible RIDα/β-transgenic mice and bred them into the MMTV-PyMT mammary tumor model, resulting in PyMT-RID^MMTV^ mice (Figure 8A). Immunostaining of the MFP with RIDβ and perilipin antibodies highlighted the mammary gland epithelial cell–specific expression of RIDβ in these mice (Figure 8B and Supplemental Figure 22; detailed images). This was evident with no detectable expression of RIDβ in adipocytes. Because of the limitations of the RIDβ antibody, which is a rabbit polyclonal antiserum, there was a slight background signal for RIDβ in mammary gland epithelial cells of PyMT mice. However, a much higher RIDβ signal was observed in such cells in PyMT-RID^MMTV^ mice (Figure 8B). We found that abnormal mammary gland development was markedly reduced in PyMT-RID^MMTV^ mice compared with PyMT mice, in contrast with our observations in PyMT-RID^ad^ mice (Figure 8C and Supplemental Figure 23; additional images). Additionally, PyMT-RID^MMTV^ mice exhibited a comparable mammary tumor onset, developing palpable tumors around the same time as PyMT mice (Figure 8D). Notably though, PyMT-RID^MMTV^ mice showed a significant reduction in tumor growth accompanied by an increase in body weight (Figure 8, E–G), demonstrating an effect opposite to that observed in PyMT-RID^ad^ mice. Lastly, CLS occurrence and TAM accumulation were comparable between PyMT-RID^MMTV^ and control mice (Figure 8, H–J, and Supplemental Figure 24; detailed images). To determine whether RIDα/β alters tumor cell proliferation in a cell-autonomous manner or through interactions with the tumor microenvironment, we performed proliferation assays using an immortalized PyMT cell line. RIDα/β overexpression dramatically reduced the proliferation of these tumor cells (Supplemental Figure 25), suggesting that cell-autonomous effects play a relevant role. Taken together, our observations indicate that the inhibition of inflammation directly in the tumor cells delays tumor growth but does not affect tumor onset or local macrophage accumulation. The manifestation of distinct phenotypes in the PyMT-RID^MMTV^ and PyMT-RID^ad^ models suggests that an inhibition of inflammatory signaling in tumor cells or distinct cell types in the tumor microenvironment has complex effects on tumor development. This underscores the critical need to better understand how inflammatory signaling in distinct cell types shapes cancer development and progression.

## Discussion

In this study, we investigated the intricate interplay of adipocyte inflammation, tumor cell inflammation, macrophage dynamics, and mammary tumor development. Our approach involved breeding RID^ad^ mice with the MMTV-PyMT mammary tumor model to generate PyMT-RID^ad^ mice. This cross-breeding facilitated the examination of a targeted suppression of inflammation in adipocytes and its specific impact on mammary tumor progression. We extended our studies by breeding RID^MMTV^ mice with the MMTV-PyMT mammary tumor model to generate PyMT-RID^MMTV^ mice. This approach enabled a comparative analysis of the impact of cell-specific inflammation suppression on mammary cancer development and progression. Through these distinct mouse models, our findings unveiled unique relationships, particularly the crucial role of the adipocyte, in determining the tumor microenvironment.

### Long-term HFD challenge of RID^ad^ mice.

Our studies using the RID^ad^ mouse model revealed a complex interplay between adipocyte inflammation, adipocyte apoptosis, and macrophage dynamics (Figures 2 and 3). The observed upregulation of macrophage-related markers in the MFP and gWAT suggests a potential link between dysregulated adipocyte inflammatory signaling and immune cell infiltration. Supporting this notion, we observed concurrent phenomena of increased adipocyte apoptosis and formation of CLSs after selective suppression of adipocyte inflammation (Figure 2). Moreover, the phenomenon of macrophage accumulation in the MFP of RID^ad^ mice was similar to that of TAMs (Figure 3), suggesting that suppression of adipocyte inflammation may create a tumor-favorable microenvironment, thereby promoting tumor growth (43–46). The significant upregulation of *Ccl2* in both the MFP and gWAT of RID^ad^ mice, along with the increased presence of macrophages that resemble TAMs, highlights the importance of *Ccl2* in driving macrophage infiltration and polarization. The observed upregulation of M2 macrophage-related genes further supports the notion of *Ccl2* promoting local macrophage polarization (47, 48), indicating a potential feedback loop between adipose tissue inflammation and immune modulation in female mice. These results highlight the multifunctional role of *Ccl2* in adipose tissue inflammation and provide insights into its impact on the tumor microenvironment. To address the observed discrepancy in the regulation of inflammatory genes between male and female mice, we investigated the cellular source of inflammatory gene induction in the MFP of RID^ad^ mice. Our findings suggest that this induction is driven by cell types other than adipocytes. Although isolated adipocytes from the MFPs of RID^ad^ mice did not exhibit increased inflammatory gene expression, the upregulation of *Ccl2* and M2 macrophage markers in whole MFPs indicates that infiltrating immune cells are the crucial contributors to the observed inflammatory environment.

### Long-term HFD challenge of PyMT-RID^ad^ mice.

Transitioning to the PyMT-RID^ad^ mouse model, our focus shifted to the MFP, considering its crucial role in breast cancer development. Strikingly, we observed that the suppression of adipocyte inflammatory signaling led to abnormal mammary gland development and significantly accelerated mammary tumor onset and growth (Figure 4). This coincided with elevated levels of Ki67-positive cells and increased blood vessel formation. Importantly, metabolic dysfunction was observed in the surrounding adipose tissue, characterized by a decrease in the uptake of fatty acids, while tumors exhibited increased expression of CD36 and enhanced fatty acid uptake, suggesting a metabolic shift toward fatty acid utilization in the tumor itself at the expense of the surrounding adipocytes (Figure 5). This metabolic shift indicated a potential redirection of lipid resources to fuel the highly proliferative tumor growth in PyMT-RID^ad^ mice.

### Short-term HFD challenge of PyMT-RID^ad^ mice.

To gain an understanding of the processes that foster early tumor development, we also explored the changes occurring in the MFP of PyMT-RID^ad^ mice at an early stage. Notably, a short-term HFD challenge of PyMT-RID^ad^ mice induced increased CLS formation, indicative of heightened adipocyte apoptosis, and macrophage recruitment (Figure 6). However, we did not detect elevated levels of cleaved caspase-3 at this stage, which may result from the still efficient and rapid clearance of dying cells by local macrophages. Full activation of caspase-3 may require prolonged signaling, becoming more evident at 12 weeks. Additionally, although *Ccl2* was significantly upregulated in the MFP of RID^ad^ mice after 12 weeks of Dox-HFD (Figure 1), it was not significantly changed in the MFP of PyMT-RID^ad^ mice after 4 weeks of Dox-HFD (Supplemental Figure 19). This phenomenon likely reflects a process in which macrophages initially aggregate around apoptotic or dysfunctional adipocytes induced by RIDα/β, gradually transitioning toward a more TAM-like phenotype and subsequently expressing *Ccl2*. This suggests that the observed differences in *Ccl2* expression between RID^ad^ and PyMT-RID^ad^ mice may be driven by the gradual accumulation and activation of macrophages over time. Importantly, despite the slight reduction in *Ccl2* expression, PyMT-RID^ad^ mice still demonstrated the recruitment and accumulation of TAMs (Figure 6), indicating that TAM recruitment emerges as a critical factor influencing early tumor growth. In our RNA-Seq analysis of the inguinal MFP of PyMT-RID^ad^ mice after 4 weeks of Dox-HFD, we discovered compelling relationships between suppressed inflammation, altered insulin signaling, and rewired cellular metabolism. Specifically, we observed a downregulation of glycolysis-related and glycogen metabolism–related gene expression (Supplemental Figure 19). These alterations coincide with lowered systemic insulin sensitivity in these mice. Pathway analyses supported the finding of impaired insulin signaling in the MFP of PyMT-RID^ad^ mice including a downregulation of the PI3K-Akt pathway (Supplemental Figure 20). Moreover, thermogenic processes within adipose tissue, including the browning of white adipocytes, relies on proper insulin signaling (49). Illustrating this dependence, E4orf1 overexpression mice display not only diminished insulin signaling but also reduced adipocyte browning (50). This suggests that disrupted insulin signaling in the MFP of PyMT-RID^ad^ mice may be a crucial driver of the observed downregulation of glycolysis, glycogen, and browning-related and “beiging”-related genes. In particular, it has been shown that reduced glycolysis is linked to decreased thermogenesis, potentially exacerbating insulin resistance (49, 51). Importantly, decreased uptake of lipids by adipose tissue due to insulin resistance may also enable tumors to take up and metabolize more lipids. This may effectively enhance tumor growth and metastasis through the upregulation of tumor progression–related and metastasis-related genes as well as genes related to mammary gland and duct morphogenesis. Interestingly, our findings also indicate that RIDα/β overexpression in adipocytes promotes an immunosuppressive tumor microenvironment through upregulation of *Ido1*, *Mrc1*, and *Cd200*. Despite no relevant changes in major immune cell populations such as M1 macrophages, DCs, NK cells, or T cells, the elevated expression of these immunoregulatory genes, particularly IDO1, suggests that RIDα/β-driven immune modulation may facilitate tumor immune escape in conjunction with TAM-mediated immune modulation.

### Long-term HFD challenge in PyMT-RID^MMTV^ mice.

Intriguingly, the suppression of tumor cell inflammation in the PyMT-RID^MMTV^ model resulted in outcomes distinct from those in the PyMT-RID^ad^ model (Figure 8). Abnormal mammary gland development was minimal, emphasizing the tumor-specific effects of inflammation inhibition. Although tumor onset remained comparable between the groups, a significant reduction in tumor growth was observed in PyMT-RID^MMTV^ mice without affecting the number of CLSs (Figure 8). The recruitment of TAMs slightly decreased, which is consistent with a reduction in tumor growth, suggesting that the accumulation of TAMs near the tumor is a critical driver of tumor progression (Figure 8). The observed reduction in abnormal mammary gland development and tumor growth highlights the potential therapeutic significance of targeting inflammation in a tumor cell–selective manner. Further investigations into the underlying mechanisms governing the recruitment of TAMs and their impact on tumor progression appears necessary. Notably, the divergent phenotypes of the PyMT-RID^ad^ and PyMT-RID^MMTV^ models underscore the cell-specific effects of the inhibition of inflammation on mammary tumor development.

Collectively, our studies using distinct mouse models provide comprehensive insight into the cell type–specific contributions of inflammation to mammary tumor development and progression. The varying outcomes observed in different models emphasize the need for a careful assessment regarding targeting inflammation for therapeutic purposes, be it in adipocytes or in tumor cells. These outcomes may ultimately also have an impact on the effectiveness of checkpoint inhibitors, which can trigger inflammation that hinders their effectiveness.

## Methods

### Sex as a biological variable.

This study exclusively examined female mice because the disease modeled, breast cancer, predominantly affects females.

### Mouse models.

All animal experimental protocols, including those for mouse use and euthanasia, were reviewed and approved by the IACUC of University of Texas Southwestern (UTSW) Medical Center under the animal protocol 2015-101207. The transgenic strains — adipocyte-specific, Dox-inducible RIDα/β-transgenic mice (*Adipoq-rtTA* × *TRE-RID*α*/*β mice or RID^ad^) — were generated by our laboratory as previously described (1, 7). We established spontaneous breast tumor development mouse models by introducing these transgenes into the *MMTV-PyMT* mammary tumor model (PyMT-RID^ad^). To establish mammary gland epithelial cell–specific, Dox-inducible RIDα/β-transgenic mice with *MMTV-PyMT* (PyMT-RID^MMTV^), RID^ad^ mice were crossed with *MMTV-rtTA/MMTV-PyMT* mice. *Adipoq-rtTA* was washed out and replaced with *MMTV-rtTA*. The transgenic strains, *MMTV-rtTA* and *MMTV-PyMT* mice, were previously generated and characterized by our laboratory (52). In each experiment, littermate mice that lacked the *TRE-RID*α*/*β transgene were chosen as the control. These control mice were fed the same diet as the experimental group carrying the *TRE-RID*α*/*β transgene (i.e., either HFD or Dox-HFD). All mice used in this study, including littermate controls, were maintained on a pure C57BL/6 genetic background. Mice were housed under barrier conditions on a 12-hour light/12-dark cycle in a temperature-controlled environment (22°C) with ad libitum access to autoclaved water and food. Cages were changed every other week, and constant veterinary supervision was provided. Diets used in this study were a regular chow diet (LabDiet, 5058), HFD (60% of calories from fat, Bio-Serv, S1850), and Dox-HFD (600 mg/kg Dox; HFD of 60% of calories from fat, Bio-Serv, S5867). Only female mice were used because females are susceptible to the development of mammary tumors.

### Genotyping PCR.

The small portion of the mouse tail tip was lysed in 100 μL of 50 mM NaOH at 95°C for 1.5 hours and then neutralized by adding 10 μL of 1M Tris-HCl (pH 8.0). After vortexing and a brief spin down, 1 μL of the supernatant was utilized as the PCR template. The primer pairs for genotyping PCR are listed in Supplemental Table 2. The PCR program consisted of an initial step at 95°C for 1 minute, followed by 30–35 cycles of 95°C for 15 seconds, 60°C for 30 seconds, and 72°C for 30 seconds, concluding with a final step at 72°C for 3 minutes. The size of the amplified DNA was confirmed using 1%–2% agarose gel electrophoresis with ethidium bromide staining.

### EO771 syngeneic breast cancer model.

To establish the EO771 syngeneic breast cancer model, EO771 cells (10^5^ cells in 50 μL, mixed in a 1:1 ratio with PBS and Matrigel) were injected subcutaneously into the MFP of 16-week-old mice that had been fed an HFD for 8 weeks. One week after cell injection, mice with subcutaneous tumors reaching approximately 100 mm³ were selected for each group and switched to a Dox-containing HFD. Tumor growth was monitored and measured using electronic calipers approximately every 2 days. Tumor volume was calculated using the modified ellipsoid formula: tumor volume = ½ (length × width²). Animals were euthanized 21 days after injection.

### Metabolic phenotyping.

Assessments of systemic metabolism, including oral glucose tolerance tests (OGTTs), insulin tolerance tests (ITTs), and triglyceride tolerance tests (TGTTs), were performed as previously described (53). For OGTTs, mice were fasted for 4–6 hours and subjected to an oral gavage of dextrose (2.5 mg/g body weight). Tail blood was collected at 0, 15, 30, 60, and 120 minutes in capillary tubes and prepared for serum and assayed for glucose. For ITTs, random-fed mice were administered insulin (1.5 U/kg Humulin R, Eli Lilly) by intraperitoneal injection. Serum glucose level was measured at 0, 15, 30, 60, and 120 minutes. For TGTTs, mice were fasted for 16 hours and subjected to an oral gavage of 20% intralipid (10 μL/g body weight, 100 mL, Sigma-Aldrich, I141). Tail blood was collected at 0, 1.5, 3, and 6 hours for triglyceride, nonesterified fatty acid, and glycerol assays. Glucose, insulin, and triglyceride levels were measured using a Contour blood glucose monitor (Bayer, 9545C), oxidase-peroxidase assay (Sigma-Aldrich), insulin ELISA (Crystal Chem), and Infinity triglycerides reagent (Thermo Fisher Scientific). Nonesterified fatty acids were measured by free fatty acid quantification kits (Wako Diagnostics, NEFA-HR2).

### Triolein uptake assay.

Triolein uptake was measured as described previously (54). Briefly, mice were fasted for 16 hours and ^3^H-triolein (PerkinElmer, NET431001MC; 2 μCi per mouse in 100 μL of 5% intralipid) was administered by retro-orbital injection. Blood samples of 150 μL were collected at 1, 2, 5, 10, and 15 minutes. At the 15-minute mark, mice were euthanized, additional blood samples were obtained, and select tissues were promptly excised, weighed, flash-frozen in liquid nitrogen, and stored at –80°C until further processing. Tissue radioactivity, including blood samples, was quantified using a Tri-Carb 2910 TR scintillation counter (PerkinElmer).

### Tissue preparation and immunostaining.

Mice were euthanized by cervical dislocation after isoflurane anesthesia. Subsequently, tissues were promptly collected and fixed in 10% buffered formalin (Thermo Fisher Scientific) for 24 hours at room temperature. Afterward, the tissues were rinsed with 50% ethanol, followed by embedding in paraffin blocks and cutting into 5 μm sections. For immunofluorescence staining, 5 μm paraffin sections were baked for 30 minutes at 60°C and deparaffinized in xylene and ethanol, followed by boiling in antigen retrieval solution (Vector Labs) containing 0.1% Tween 20. Slides were blocked in blocking buffer consisting of 10% goat serum (Thermo Fisher Scientific) in PBS-T (0.1% Tween 20 in PBS) for 1 hour at room temperature and then overnight at 4°C with the primary antibodies RIDβ (made in-house), perilipin (20R-PP004, Fitzgerald), cleaved caspase-3 (9661, Cell Signaling Technology), MAC-2 (125401, BioLegend), CD163 (16646-1-AP, ProteinTech), Ki67 (ab15580, Abcam), CD31 (14-0311-82, Invitrogen), CD36 (PA1-16813, Invitrogen), FABP4 (PA5-30591, Invitrogen), and ERα (made in-house). The next day, slides were washed 5 times with PBS-T and incubated with secondary antibodies labeled with Alexa Fluor 488 (A-11006, Thermo Fisher Scientific) or Alexa Fluor 594 (A-11037, Thermo Fisher Scientific) diluted in blocking buffer for 1 hour at room temperature. Slides were washed 5 times with PBS-T, and then they were mounted with Vectashield mounting medium with DAPI (Vector Labs). Immunofluorescence staining was imaged using a Zeiss LSM880 confocal microscope and analyzed by Fiji/ImageJ (NIH; version 2.1.0/1.53h). Image quantification was performed by merging the channel of the protein of interest with DAPI. Cells positive for the protein of interest were then counted and expressed as a percentage of the total nuclei. For CD31, the positive area was measured as a percentage of the total area in the field. All image quantifications were performed using 2–3 mice per group. H&E staining was performed using standard reagents (Abcam). All details for antibodies and reagents are listed in Supplemental Table 3.

### Tumor dissociation and primary tumor cell preparation.

Approximately 0.5 mg of tumor tissue was excised from euthanized mice and finely minced on a sterile surface. The minced tissue was incubated in a digestion buffer containing collagenase B, collagenase D, and DNase I at 37°C with gentle stirring for 15–30 minutes. After enzymatic digestion, the cell suspension was filtered through a 70 μm cell strainer, centrifuged, washed, and treated with red blood cell lysis buffer. Cells were then resuspended in DMEM supplemented with 10% FBS and 10% horse serum, and subsequently subcultured. Differential trypsinization was used to separate tumor cells from nontumorigenic cells during passaging.

### Adipocyte size measurement.

For adipocyte size quantification, bright-field H&E-stained images were acquired using a NanoZoomer S60 (Hamamatsu). Adipocyte size analysis was performed according to previously validated protocols with minor modifications (55). Fiji/ImageJ (NIH) software was used to analyze and calculate the area of each adipocyte. At least 200 adipocytes were quantified for each individual mouse.

### Isolation of floated adipocytes, stromal vascular fraction cells, and generation of in vitro–differentiated adipocytes.

The MFP of 4- to 6-week-old female mice was minced and digested for 1 hour at 37°C in a shaking water bath in HBSS (Sigma-Aldrich) containing 1.5% BSA (Sigma-Aldrich) and 1 mg/mL collagenase D (Roche). Samples were mixed every 20 minutes by gently inverting the tube to ensure uniform tissue digestion. The resulting dispersed tissue was filtered through a 100 μm cell strainer and centrifuged for 5 minutes at 600*g*, 4°C. Floating adipocytes were collected, and the pelleted stromal vascular fraction (SVF) cells were resuspended in culture media (DMEM/F12 containing 10% FBS, 1× GlutaMax, 1× penicillin/streptomycin, and 1× gentamicin), filtered through a 45 μm cell strainer, and subjected to a second round of centrifugation. The pelleted cells were resuspended in culture media and grown at 37°C in 5% CO_2_. For in vitro differentiation experiments, SVF cells were grown to approximately 95% confluency, and adipogenesis was induced using culture media supplemented with 500 μmol/L 3-isobutyl-1-methylxanthine, 1 μmol/L dexamethasone, 5 μg/mL insulin, and 1 μmol/L rosiglitazone for 2 days. After induction, the cells were maintained in media containing only 5 μg/mL insulin and were used for harvesting the CM at 6–8 days of differentiation.

### Preparation of CM.

Serum-free CM was collected from in vitro–differentiated adipocytes from each group of mice. The collected CM was centrifuged at 500*g* at 4°C for 10 minutes to remove any cellular debris. The supernatant was saved, aliquoted, flash-frozen in liquid nitrogen, and stored at –80°C.

### Cancer cell culture.

Human breast cancer cell lines MCF-7, MDA-MB-231, HCC-1954, and HCC38 were sourced from ATCC. Mouse breast cancer cell lines, including Met1 and EO771, were provided by Rolf Brekken at UTSW Medical Center. All cells were maintained in RPMI (Gibco) supplemented with 10% FBS (GeminiBio) and 1% penicillin/streptomycin (Sigma-Aldrich). All details for cell lines and reagents are listed in Supplemental Table 3.

### Cell proliferation assays.

Cell proliferation assays were performed as described previously (56, 57). Briefly, human and mouse breast cancer cells were plated and grown in CM (see below) harvested from in vitro–differentiated adipocytes from each group of mice. The CM was replenished every other day for the specified durations before assessing cell proliferation. Cell proliferation was assessed every 2 days through a crystal violet staining assay. To this end, the cells were washed with PBS, fixed with 5% formaldehyde for 10 minutes at room temperature, and stored in PBS at 4°C until all time points had been collected. The fixed cells were stained with a 0.1% crystal violet in a 20% methanol solution. After washing to remove unincorporated stain, the crystal violet was extracted using 10% glacial acetic acid and the absorbance was read at 595 nm.

### Protein extraction and Western blotting.

Frozen tissues were crushed into a fine powder using a tissue pulverizer, ensuring that the tissues remained frozen during the process. The frozen powder was transferred to a glass Dounce homogenizer, then resuspended in RIPA buffer (10 mM Tris-HCl, pH 8.0; 1 mM EDTA; 0.5 mM EGTA; 1% Triton X-100; 0.1% sodium deoxycholate; 0.1% SDS; 140 mM NaCl; Pierce) was added, and the tissue was disrupted with the Dounce homogenizer (10–20 times on ice). The mixture was transferred into a tube and incubated with gentle mixing for 15 minutes at 4°C. The mixture was subjected to centrifugation at maximum speed multiple times in a microcentrifuge for 10 minutes at 4°C to remove the insoluble material, and the supernatant was collected. Protein concentrations were determined using a BCA protein assay (Pierce). For Western blotting, 20 μg of protein was separated on a 4%–12% gradient polyacrylamide-SDS gel (Invitrogen) and transferred onto a nitrocellulose membrane (Bio-Rad). The membranes were blocked for 1 hour at room temperature in TBS with 0.1% Tween 20 (TBS-T) containing 5% nonfat dry milk. Primary antibodies RIDβ (rabbit polyclonal; made in-house), cleaved caspase-3 (9661, Cell Signaling Technology), GAPDH (MA5-35235, Invitrogen), and β-actin (4970, Cell Signaling Technology) were diluted in 5% nonfat dry milk (Thermo Fisher Scientific) and incubated with membranes overnight at 4°C with gentle mixing. After extensive washing with TBS-T, the membranes were incubated with an appropriate HRP-conjugated secondary antibody (Thermo Fisher Scientific) diluted in 1% nonfat dry milk for 1 hour at room temperature. The membranes were washed extensively with TBS-T before chemiluminescent detection using SuperSignal West Pico substrate (Thermo Fisher Scientific) and an iBright 1500 system (Invitrogen). All details for antibodies and reagents are listed in Supplemental Table 3.

### RNA extraction and qPCR.

Total RNA was extracted from tissues using the RNeasy Mini kit (QIAGEN), TRIzol (Invitrogen), and EZ-10 DNAaway RNA miniprep kit (Bio Basic), and cDNA pools were generated by reverse transcription using PrimeScript RT Master Mix (Takara). qPCR was performed using Powerup SYBR Green PCR Master Mix (Applied Biosystems) on a QuantStudio 6 Flex Real-Time PCR System (Applied Biosystems). Primer sequences for qPCR are listed in Supplemental Table 1.

### RNA-Seq and bioinformatic analysis.

RNA-Seq was performed by Novogene as described previously (53). Briefly, RNA isolated from the MFP or tumors was used to prepare an RNA-Seq library by enrichment from total RNA using oligo(dT) beads. For bioinformatic analysis, Novogene provided basic RNA-Seq analysis, including the list of differentially regulated genes, pathway analysis, and a heatmap. Differential gene expression analysis employed DESeq2, applying criteria of adjusted *P* value of 0.05 or less and absolute log_2_ fold change (|log_2_FoldChange|) of 1.0 or greater to identify a significantly differentially expressed gene. The volcano plot was generated using GraphPad Prism version 10.4.2 with genes from the entire gene list, including differentially regulated genes between 2 samples. Individual gene data expressed as mean ± SEM were obtained using FPKM or log_2_(FPKM) from RNA-Seq. Gene Set Enrichment Analysis (GSEA) was performed using the GSEA module available in Gene Pattern (version 20.4.0; Broad Institute; https://www.genepattern.org/). The Hallmark gene sets from the Molecular Signatures Database (MsigDB v2024.1, Broad Institute) were used to identify enriched pathways. GSEA was conducted with default settings, with minor modifications, including weighted scoring, phenotype-based permutations (1,000 permutations), gene ranking using the2-tailed Student’s *t* test, and standard normalization methods.

### Statistics.

All data are expressed as mean ± SEM. Differences between 2 groups were examined for statistical significance with an unpaired 2-tailed Student’s *t* test. Two-way ANOVA with a multiple-comparison test using GraphPad Prism software was applied to multiple time point comparisons. *P* values and adjusted *P* values for RNA-Seq were determined by use of the DESeq2 R package. A *P* value less than 0.05 denotes a statistically significant difference. Each statistics methodology used is described in the respective figure legend.

### Study approval.

All animal procedures were approved by the IACUC of UTSW Medical Center (APN2015-101207).

### Data availability.

All data supporting the findings of this study are available within the article and its supplemental materials, including source data. RNA-Seq data are available in the NCBI’s Gene Expression Omnibus (GEO GSE276790). Values for all data points in graphs are reported in the Supporting Data Values file.

## Author contributions

PES conceived the project. DSK and PES designed the experiments and supervised their implementation. DSK and TO performed most mouse-based experiments together. DSK conducted all cell-based experiments. QZ and MK provided RID^ad^ mice and intellectual input for this manuscript. SC conducted the triolein uptake assay with assistance from DSK and TO. JBF, KM, MK, CJ, and C Li provided assistance in interpreting the results and with mouse-related tasks. QL and KM performed adipocyte measurement experiments. C Lee, JV, and MV assisted with histology. DSK prepared the initial drafts of the figures and text with the help of TO, which were then reviewed and edited by JBF, RMW, and PES. The manuscript was finalized by PES. PES is the guarantor of this work and had full access to all the data in the study, taking responsibility for its integrity and the accuracy of the analysis. PES secured funding for the project and provided intellectual support across all aspects of the work. DSK and TO contributed equally to the conducted experiments and are recognized as co–first authors. The order of the co–first authors was based on the roles of the authors; DSK was listed first because he initiated the project, designed the experiments, and drafted the original manuscript**.**

## Supplementary Material

Supplemental data

Unedited blot and gel images

Supporting data values

## Version 1 06/17/2025

In-Press Preview

## Version 2 08/15/2025

Electronic publication
