# Supplementary material for: Inhibiting inflammation in adipocytes accelerates mammary tumor development in mice
Source: J Clin Invest. 2025 Jun 17;135(16):e187202. doi: 10.1172/JCI187202 (PMC12352902; doi:10.1172/JCI187202)
Supplement: Supplemental data [file jci-135-187202-s286.pdf]

SUPPLEMENTAL INFORMATION

Inhibiting inflammation in adipocytes accelerates mammary tumor development in mice

Dae-Seok Kim[1], Toshiharu Onodera[1,2], Jan-Bernd Funcke[1], Kyounghee Min[1], Qingzhang Zhu[1], Qian Lin, Shiuhwei Chen[1], Chanmin Joung[1], Min Kim[3], R. Max Wynn[1], Joselin Velasco[1], Charlotte Lee[4], Megan Virostek[1], Chao Li [1], and Philipp E Scherer[1,*]

[1] Touchstone Diabetes Center, The University of Texas Southwestern Medical Center, Dallas, TX, United States

[2] Department of Adipose Management, Osaka University Graduate School of Medicine, Osaka, Japan

[3] Department of Biological Sciences, School of Life Sciences, Ulsan National Institute of Science and Technology, Ulsan, South Korea

[4] Center for Hypothalamic Research, Department of Internal Medicine, The University of Texas Southwestern Medical Center, Dallas, TX, United States

<u>List of Supplemental Information:</u>

Supplemental Figures 1-25

Supplemental Table 1-3

* Corresponding author: Philipp E Scherer, Ph.D.
Touchstone Diabetes Center, The University of Texas Southwestern Medical Center, Dallas, United States.
E-mail : Philipp.Scherer@UTSouthwestern.edu
Tel : +1-214-648-8715 Fax : (214) 648-8720

Authorship notes: D-SK and TO are co-first authors and contributed equally to this work.

Conflict of interest: The authors have declared that no conflict of interest exists.

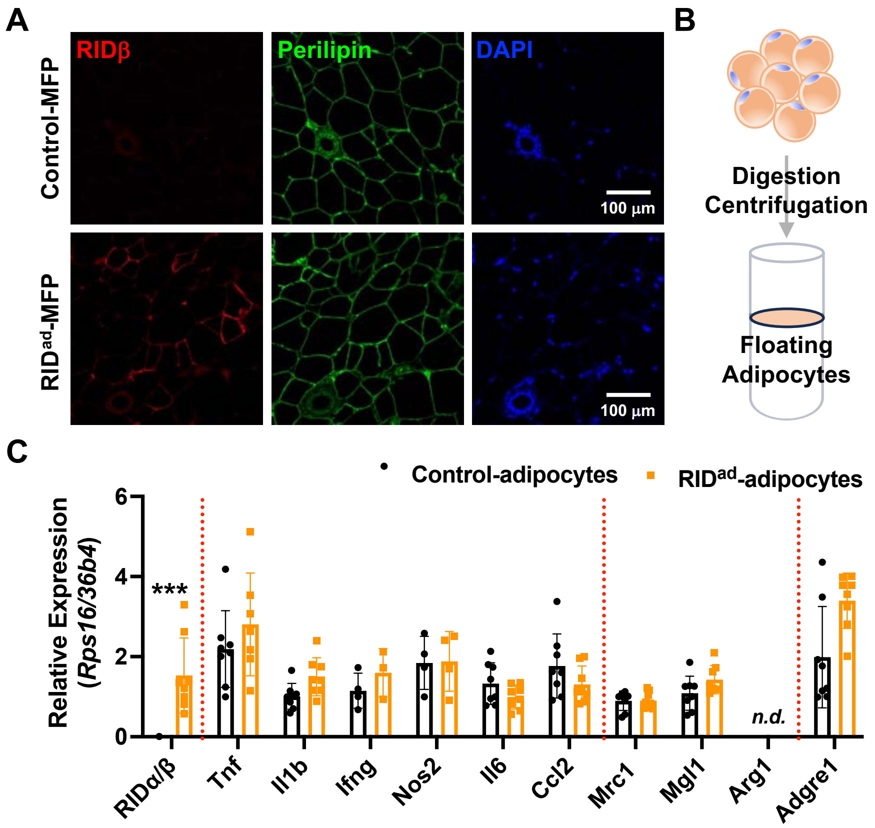

Supplemental Figure 1. RIDα/β and inflammatory gene expression in adipocytes of RID^{ad} mice.

(A) Representative immunostaining of RIDβ and perilipin in the mammary fat pad (MFP). Related to Figure 1E. **(B)** Schematic of the experimental procedure for the isolation of floated adipocytes from adipose tissues. **(C)** qPCR analysis of inflammation-related mRNA expression in isolated adipocytes. *Rps16* and *36b4* were used for normalization. $n = 8$/group. **(Statistics)** (C) Data are displayed as mean±SEM and were analyzed by unpaired 2-tailed t-tests. *** *p=0.0004*

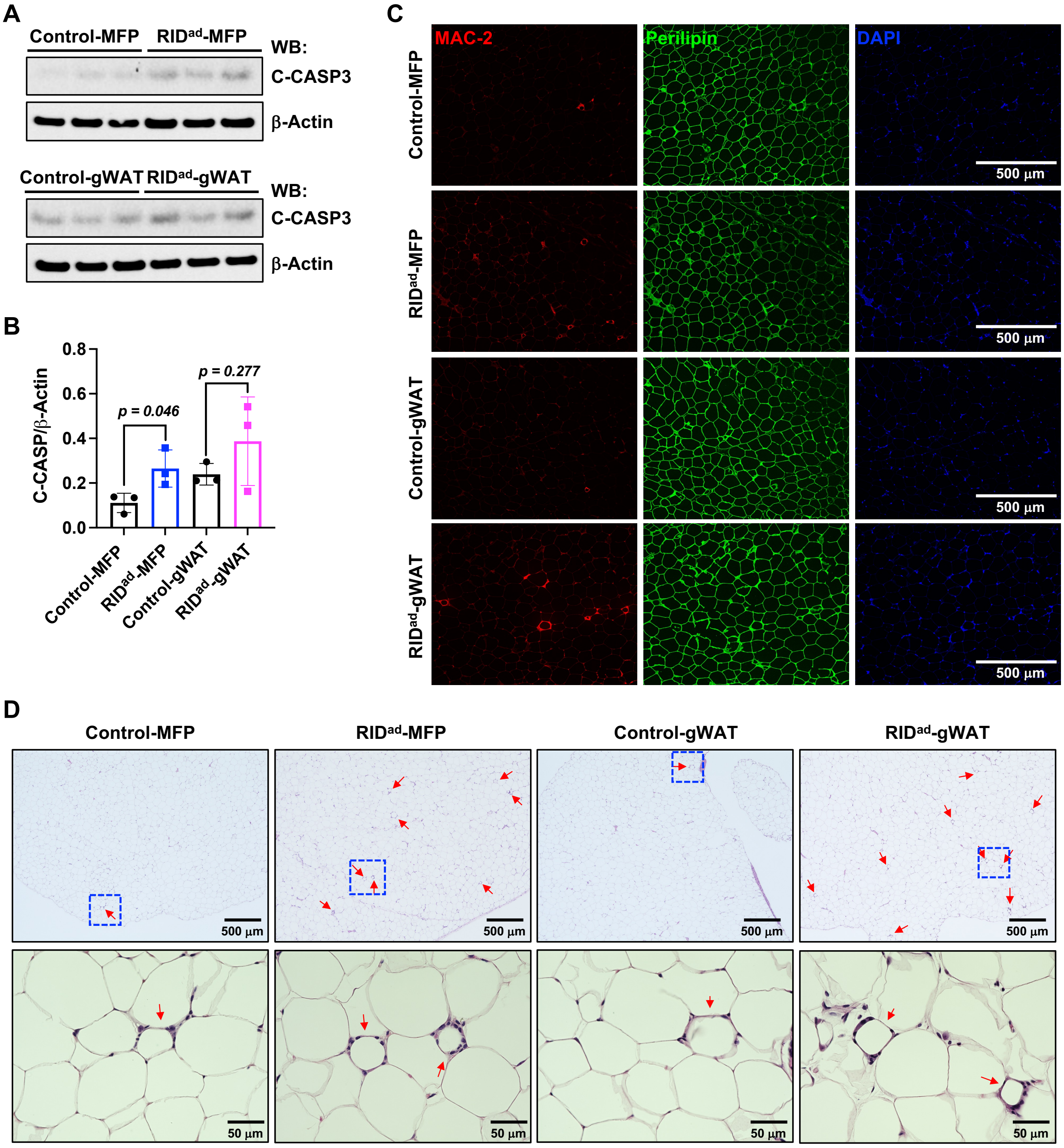

Supplemental Figure 2. Increased levels of cleaved caspase-3 and CLS staining in the MFP of RID^{ad} mice. **(A)** Western blot analysis of cleaved caspase-3 (C-CASP3) and β-Actin protein expression in the mammary fat pad (MFP) and gonadal white adipose tissue (gWAT). n = *3*/group. **(B)** Quantification of C-CASP3 protein expression from the experiments shown in (A). **(C)** Representative immunostaining of MAC-2 and perilipin in the MFP and gWAT. Related to Figure 2E and F. n = *4–5*/group. **(D)** H&E staining shows CLSs (red arrows) formed in the MFP and gWAT. **(Statistics)** (C) Data are displayed as mean±SEM and were analyzed by unpaired 2-tailed t-tests.

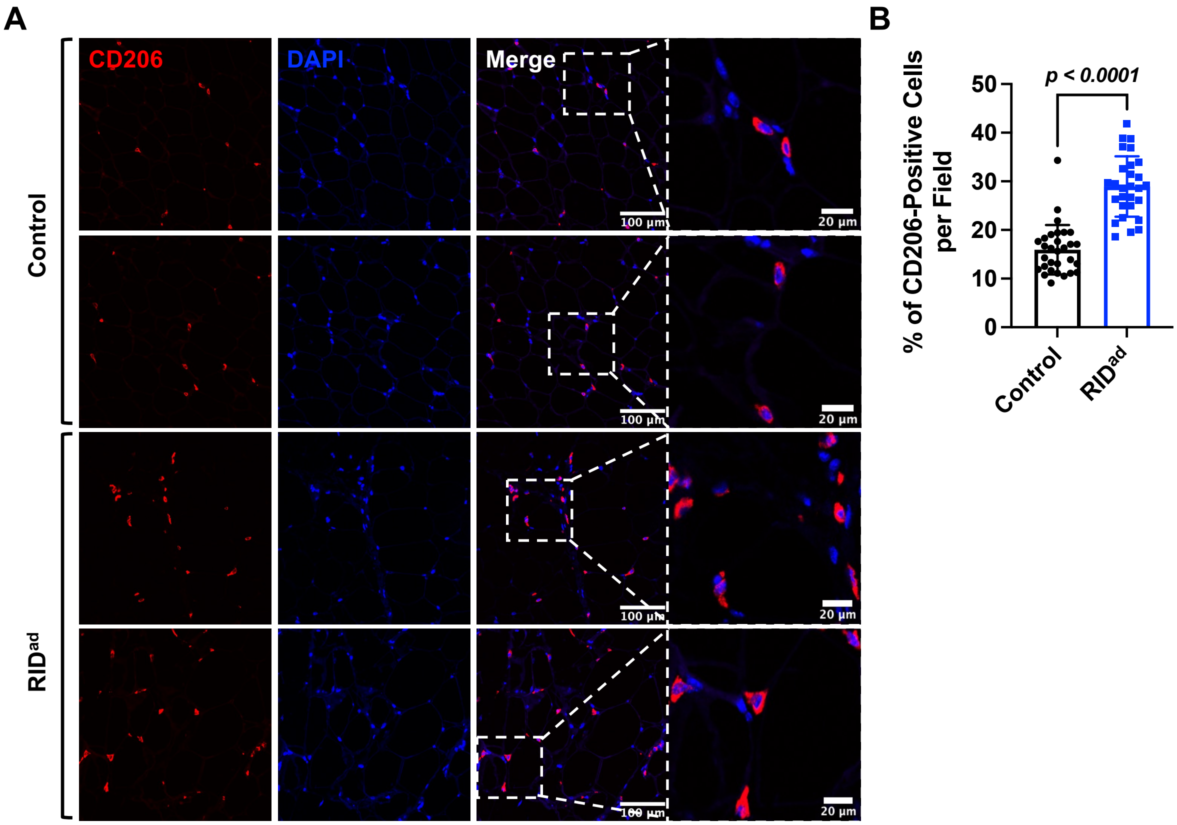

Supplemental Figure 3. Enhanced TAM infiltration into the MFP of RID^{ad} mice.
(A) Representative immunostaining of CD206 in the mammary fat pad (MFP). *n* = 2/group. **(B)** Quantification of the percentage of CD206-positive from the experiments shown in (A). **(Statistics)** (B) Data are displayed as mean±SEM and were analyzed by unpaired 2-tailed t-tests. A total of 30 images from 2 control mice and 29 images from 2 RID^{ad} mice were quantified.

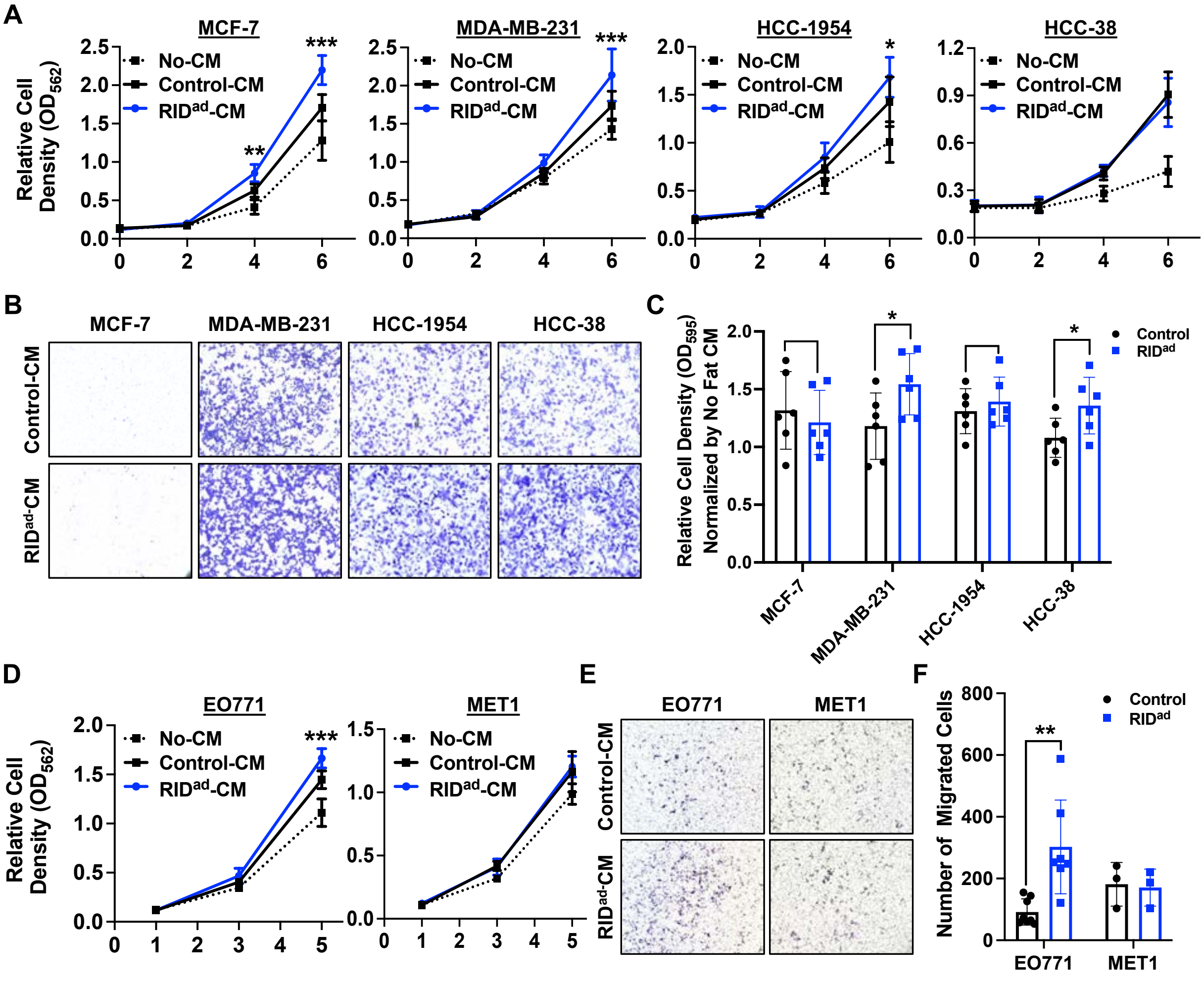

Supplemental Figure 4. Conditioned medium from *in vitro*-differentiated RID^{ad} adipocytes stimulates breast cancer cell proliferation and migration.

(A) Human breast cancer cell proliferation assays using conditioned medium (CM) from *in vitro*-differentiated adipocytes. *n = 6*/group. **(B)** Human breast cancer cell migration assays **(C)** Quantification of migration from the experiments shown in (B). *n* = 6/group. **(D)** Mouse breast cancer cell proliferation assays. *n = 6*/group. **(E)** Mouse breast cancer cell migration assays. **(F)** Quantification of migration from the experiments shown in (E). *n = 3–8*/group. **(Statistics)** (A, C, D, F) Data are displayed as mean±SEM and were analyzed by two-way ANOVA (A, D) or unpaired 2-tailed t-tests (C, F). *$p<0.01$, **$p<0.005$, ***$p<0.0001$.

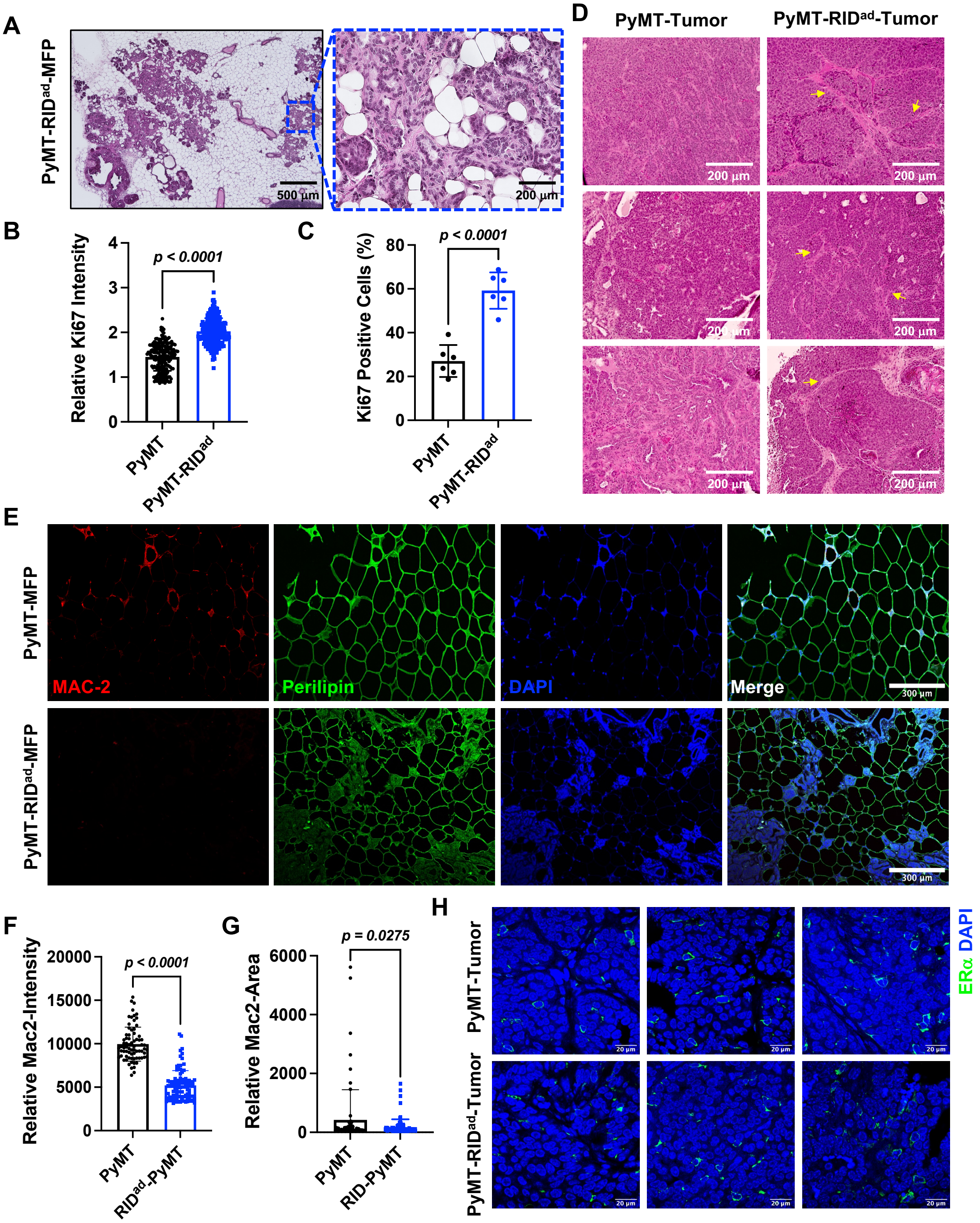

Supplemental Figure 5. Distinct phenotypes in adipocyte-specific, doxycycline-inducible PyMT-RID^{ad} mice. **(A)** H&E staining reveals abnormalities in mammary gland development in PyMT-RID^{ad} mice. **(B and C)** Quantification of Ki67 intensity (B) and percentage of Ki67-positive cells (C) from the experiments shown in Figure 4G. *n* = *2*/group. **(D)** H&E staining reveals significantly increased blood vessel formation (yellow arrows) in the mammary fat pad (MFP) of PyMT-RID^{ad} mice. *n* = *3*/group. **(E)** Representative immunostaining of MAC-2 and perilipin in the MFP **(F and G)** Quantification of MAC-2 intensity and area from the experiments shown in (E). **(H)** Representative immunostaining of estrogen receptor alpha (ERα) in the formed tumors. *n* = *3*/group. **(Statistics)** (B, C, F, G) Data are displayed as mean±SEM and were analyzed by unpaired 2-tailed t-tests.

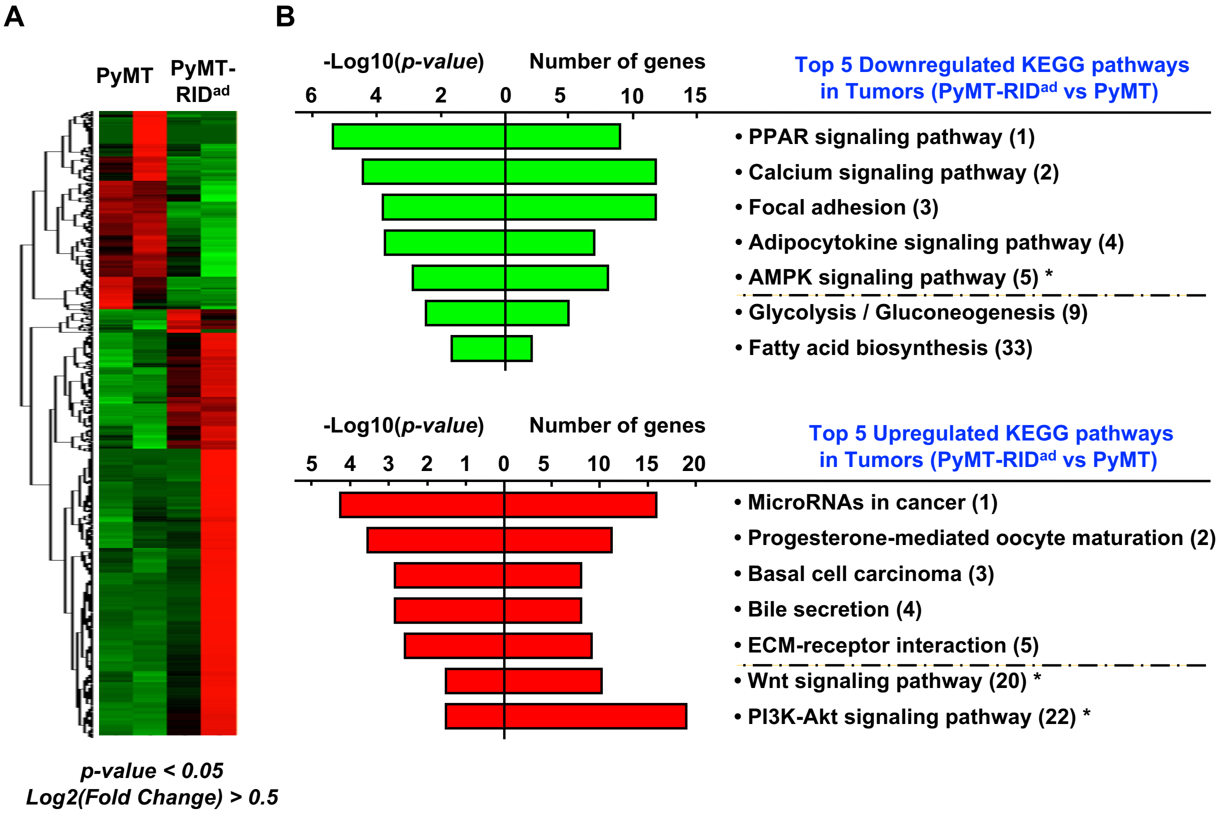

Supplemental Figure 6. Differentially expressed genes in tumors from PyMT-RID^{ad} mice compared to PyMT mice.

(A) Heatmap of differentially expressed genes by RNA-seq in tumors. *n* = 2/group **(B)** Analysis showing KEGG pathways that are downregulated (green) or upregulated (red) in the tumors from PyMT-RID^{ad} compared to PyMT mice.

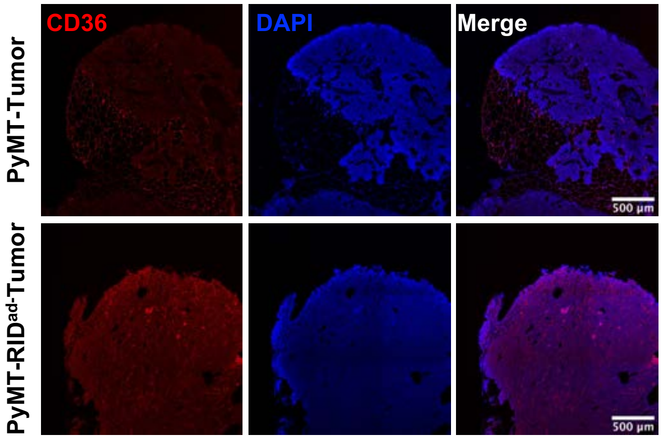

Supplemental Figure 7. CD36 is highly expressed in the tumors from PyMT-RID^{ad} mice compared to PyMT mice. Representative immunostaining of CD36 in tumors.

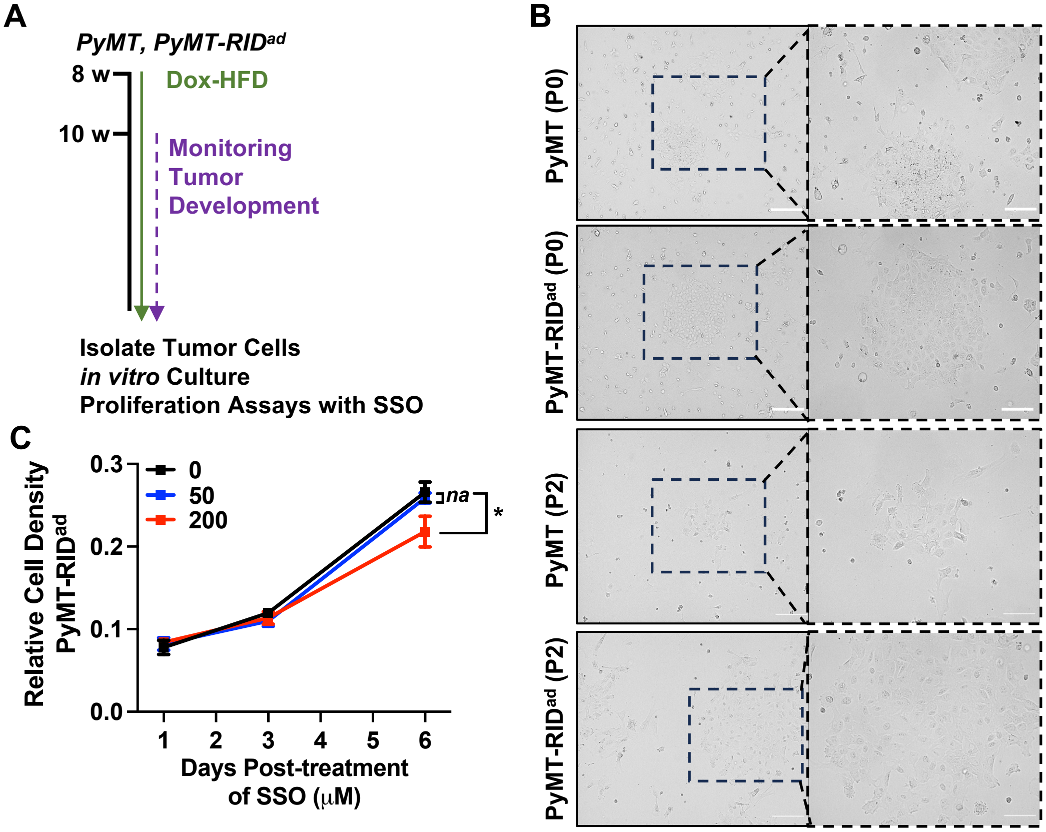

Supplemental Figure 8. Inhibition of CD36 reduces the growth of isolated tumor cells from PyMT-RID^{ad} mice.

(A) Schematic representation of the experimental design. **(B)** Representative images of isolated tumor cells at passage 0 (P0) and passage 2 (P2). *n* = *2*/group. **(C)** Proliferation assays of isolated tumor cells treated with the CD36 inhibitor SSO. *n* = *2*/group. **(Statistics)** (C) Data are displayed as mean±SEM and were analyzed by unpaired 2-tailed t-tests. *$p<0.01$.

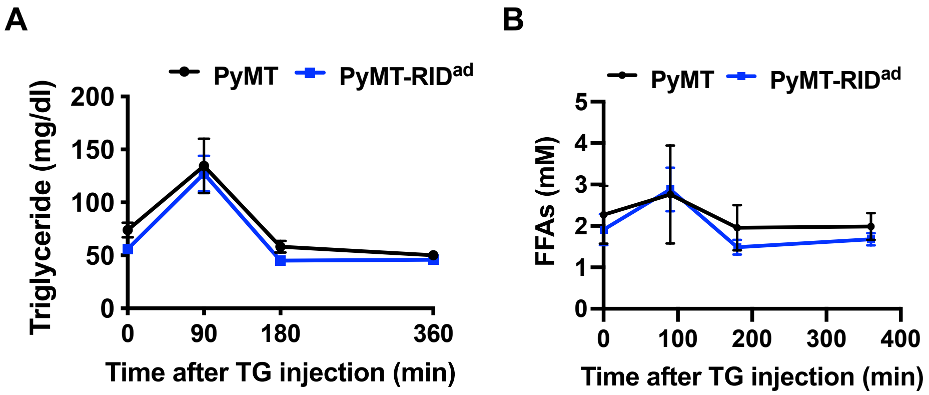

Supplemental Figure 9. Fasting plasma TG and FFAs levels in PyMT-RID^{ad} mice compared to PyMT mice.

(A and B) Oral triglyceride tolerance test (TGTT) with measurements of triglyceride (TG; A) and free fatty acid (FFA; B) levels. n = *5–6*/group. **(Statistics)** (A, B) Data are displayed as mean±SEM and were analyzed by unpaired 2-tailed t-tests. No statically difference.

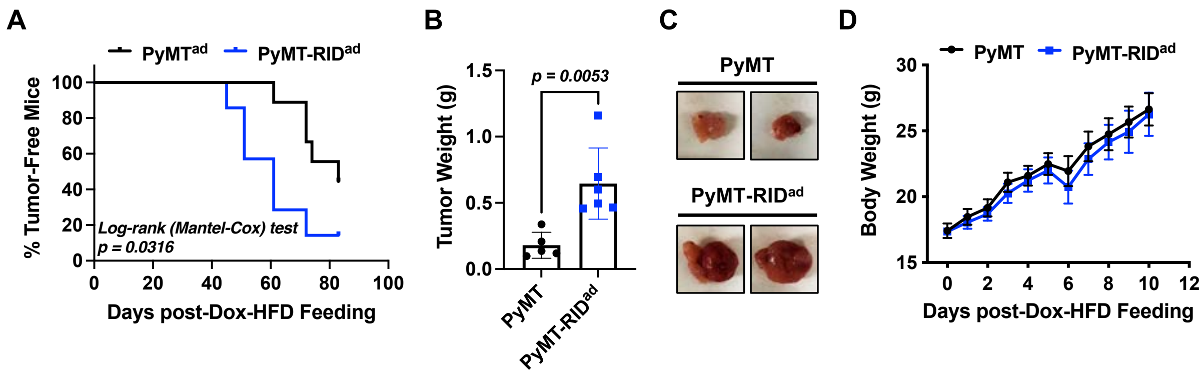

Supplemental Figure 10. Earlier mammary tumor onset and accelerated growth in PyMT-RID^{ad} mice.
(A) Kaplan-Meier tumor-free mouse curves. *n* = 7–*9*/group. **(B)** Tumor weight at the end of the experiment. *n* = 7–*9*/group. **(C)** Representative mages of tumors formed in each group at the end of the experiment. **(D)** Body weight. *n* = 7–*9*/group. **(Statistics)** (A, B, D) Data are displayed as mean±SEM and were analyzed by unpaired 2-tailed t-tests.

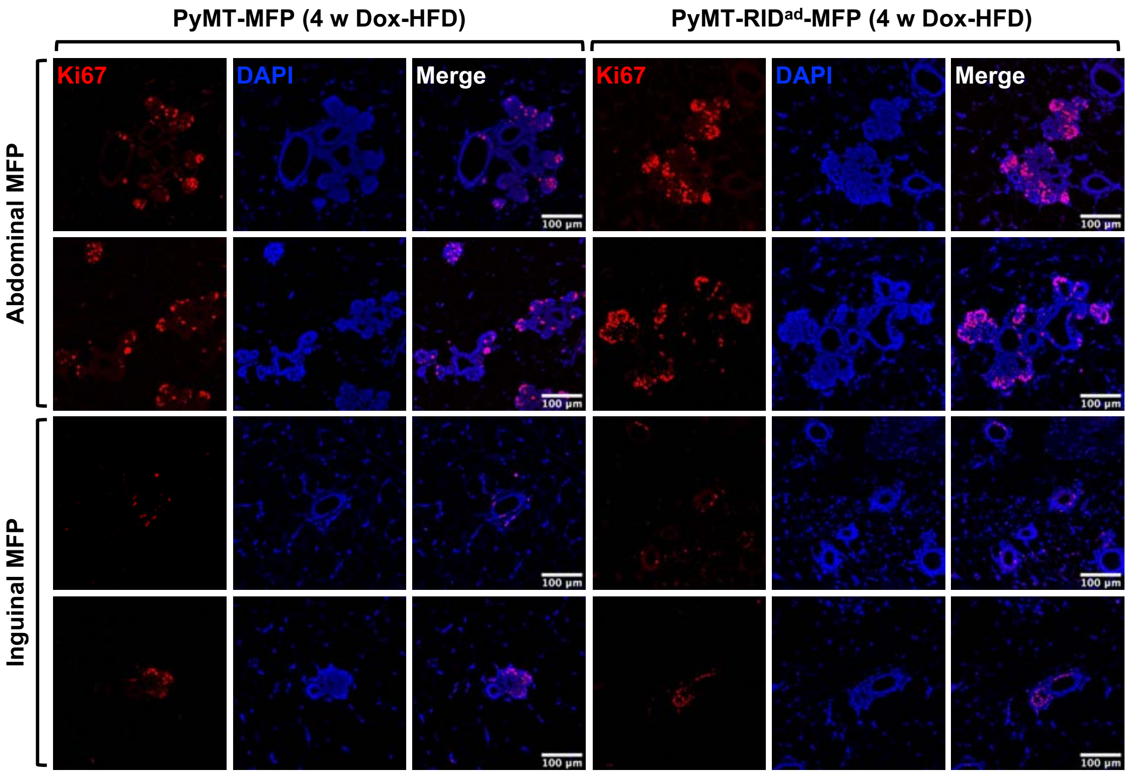

Supplemental Figure 11. Immunostaining of Ki67 in the MFP.
Representative immunostaining of Ki67 in the mammary fat pad (MFP), including both abdominal and inguinal regions**.** *n* = *2*/group.

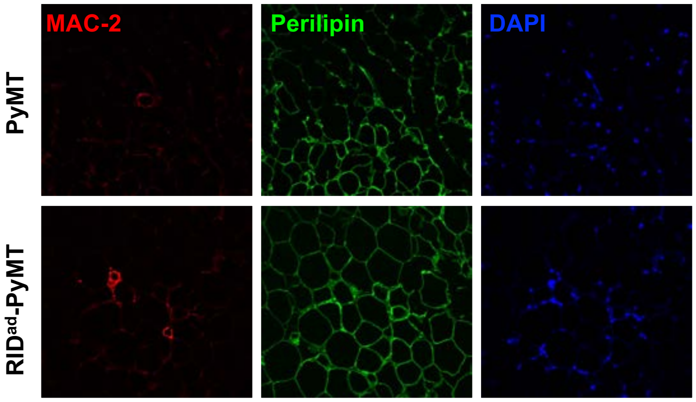

Supplemental Figure 12. Immunostaining for MAC-2 in the MFP.

Representative immunostaining of MAC-2 and perilipin in the mammary fat pad (MFP). n = 2/group. Related to Figure 6C.

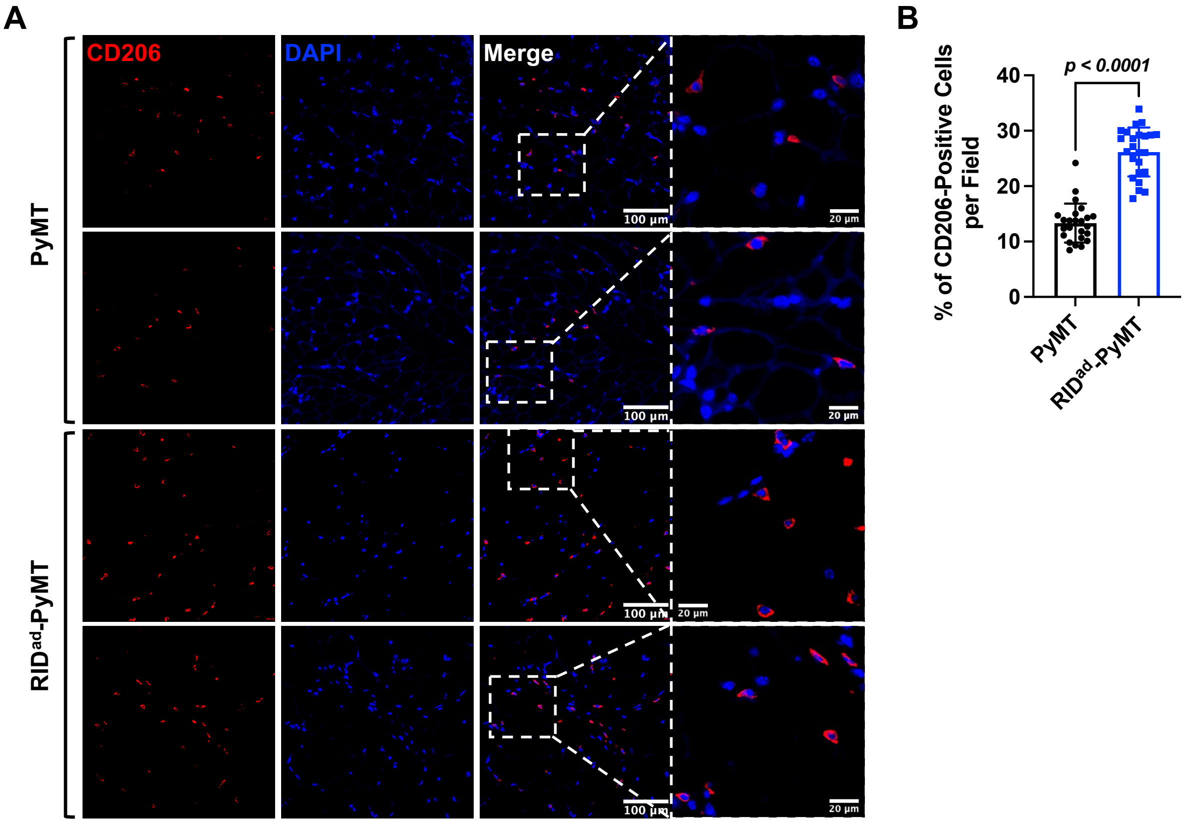

Supplemental Figure 13. Enhanced TAM infiltration into the MFP of PyMT-RID^{ad} mice. **(A)** Representative immunostaining of CD206 in the mammary fat pad (MFP). n = 2/group. **(B)** Quantification of the percentage of CD206-positive cells from the experiments shown in (A). n = 2/group. **(Statistics)** (B) Data are displayed as mean±SEM and were analyzed by unpaired 2-tailed t-tests. A total of 24 images from 2 control mice and 2 RID^{ad} mice were quantified.

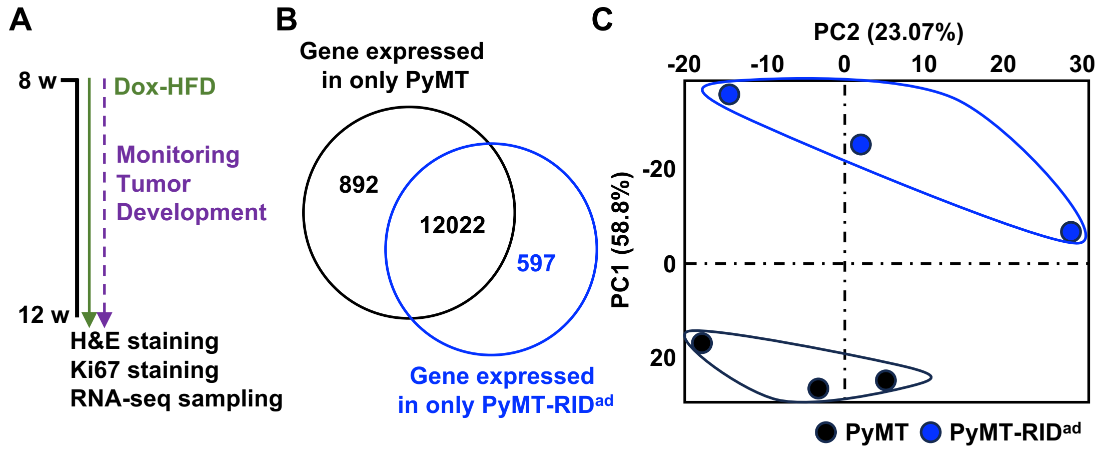

Supplemental Figure 14. Differentially expressed genes in the MFP of PyMT-RID^{ad} mice compared to PyMT mice.

(A) Schematic representation of the experimental design for RNA sampling to avoid tumor contamination. **(B)** Venn diagram showing the number of genes that are differentially expressed in the mammary fat pad (MFP) in each group of mice. *n* = *3*/group. **(C)** Principal component analysis of transcriptional signatures. *n* = 3/group.

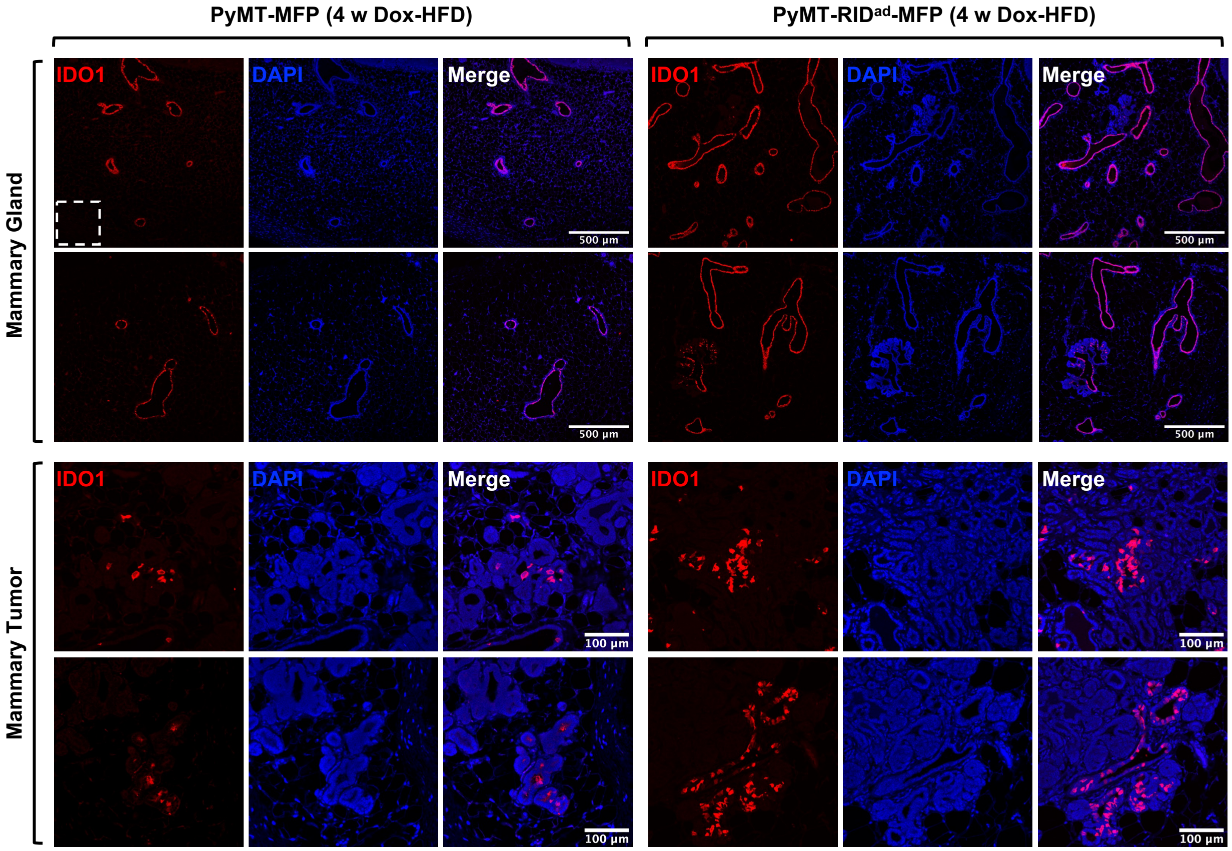

Supplemental Figure 15. Differentially expressed immunoregulatory molecules in the MFP of PyMT-RID^{ad} mice compared to PyMT mice.

Representative immunostaining of IDO1 in the mammary fat pad (MFP). *n* = 2/group.

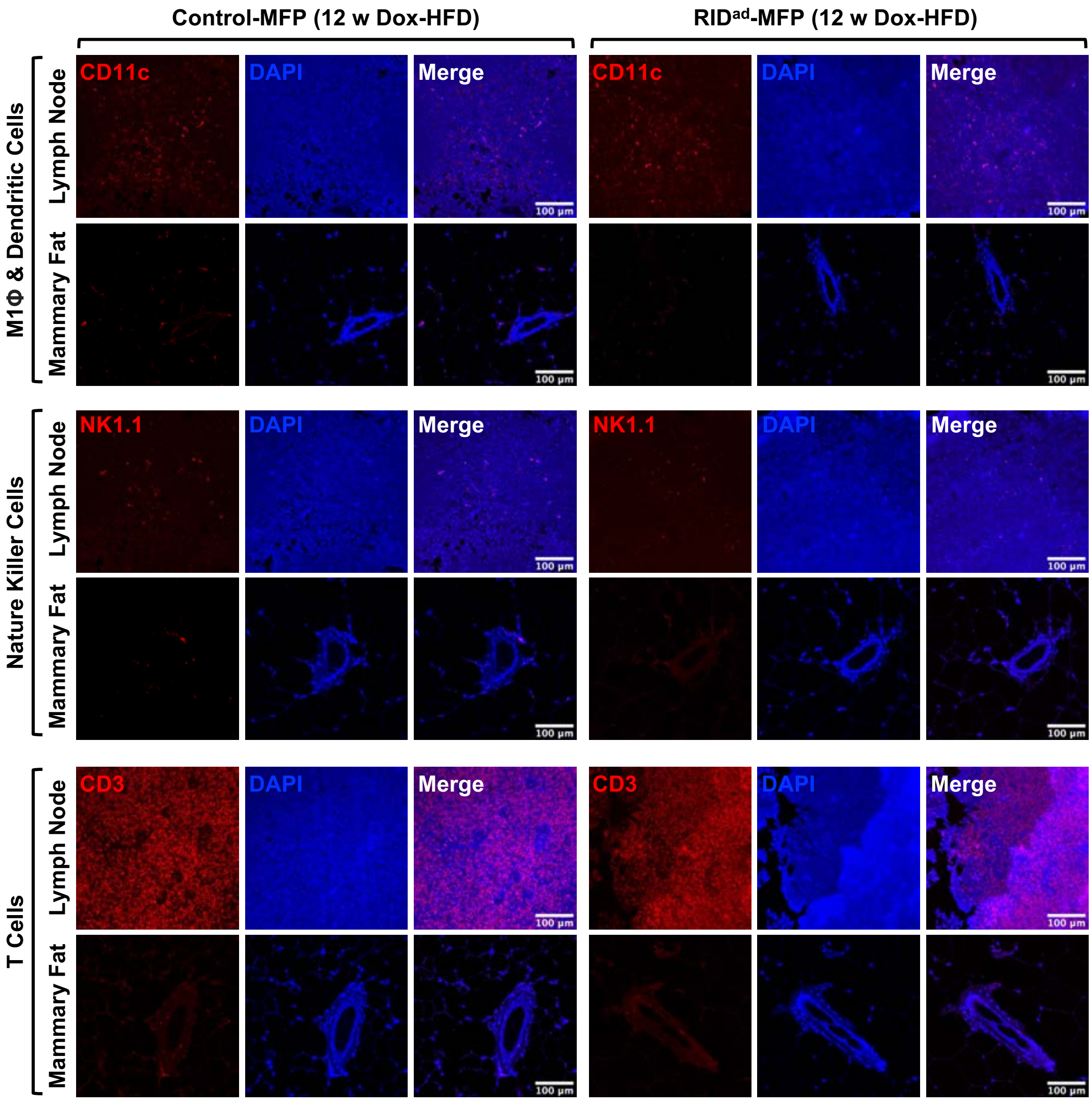

Supplemental Figure 16. No detectable differences in M1 macrophage, dendritic cell, NK cell, or T cell populations in the MFP of RID^{ad} compared to control mice. Representative immunostaining for each marker gene. *n* = 2/group.

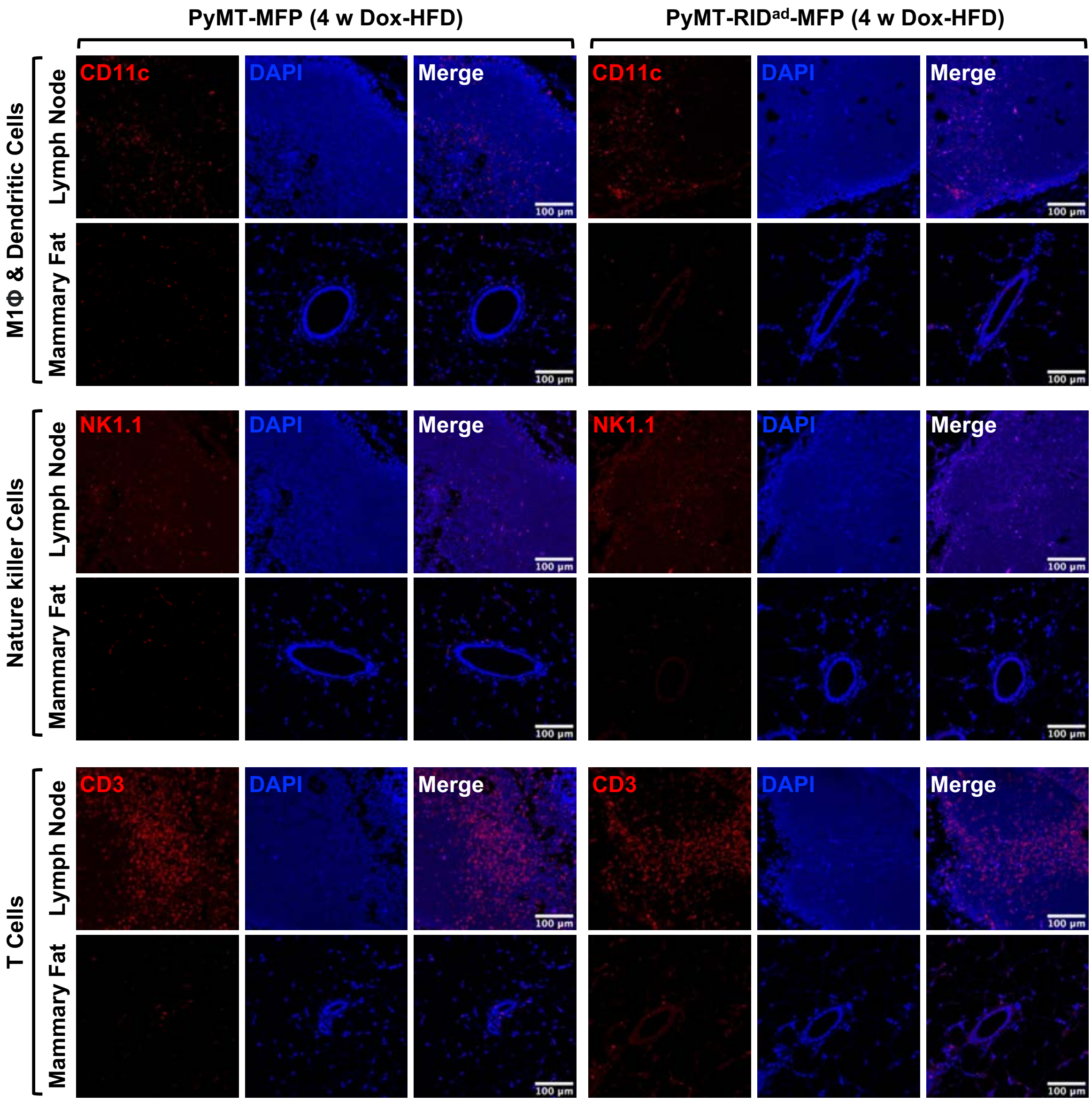

Supplemental Figure 17. No detectable differences in M1 macrophage, dendritic cell, NK cell, or T cell populations in the MFP of PyMT-RID^{ad} compared to PyMT mice.. Representative immunostaining for each marker gene. *n* = 2/group.

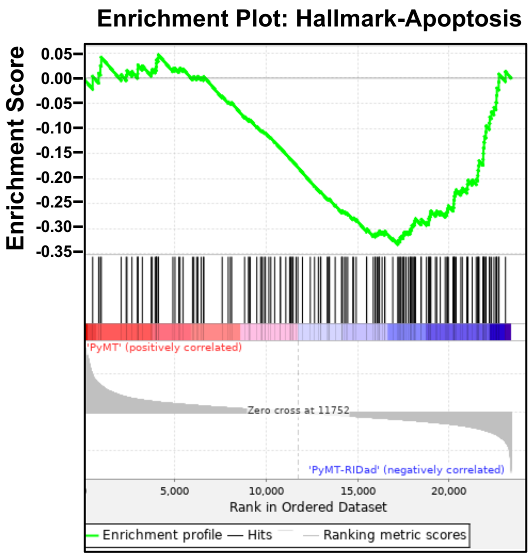

Supplemental Figure 18. Apoptotic gene enrichment and expression in PyMT-RID^{ad} mice.

GSEA enrichment plot showing that Hallmark Apoptosis gene sets are significantly enriched in the PyMT-RID^{ad} group, as indicated by the negative enrichment score (ES) and their accumulation on the negative side of the ranked list metric (ES = -0.332, NES = -1.556, $p < 0.0001$).

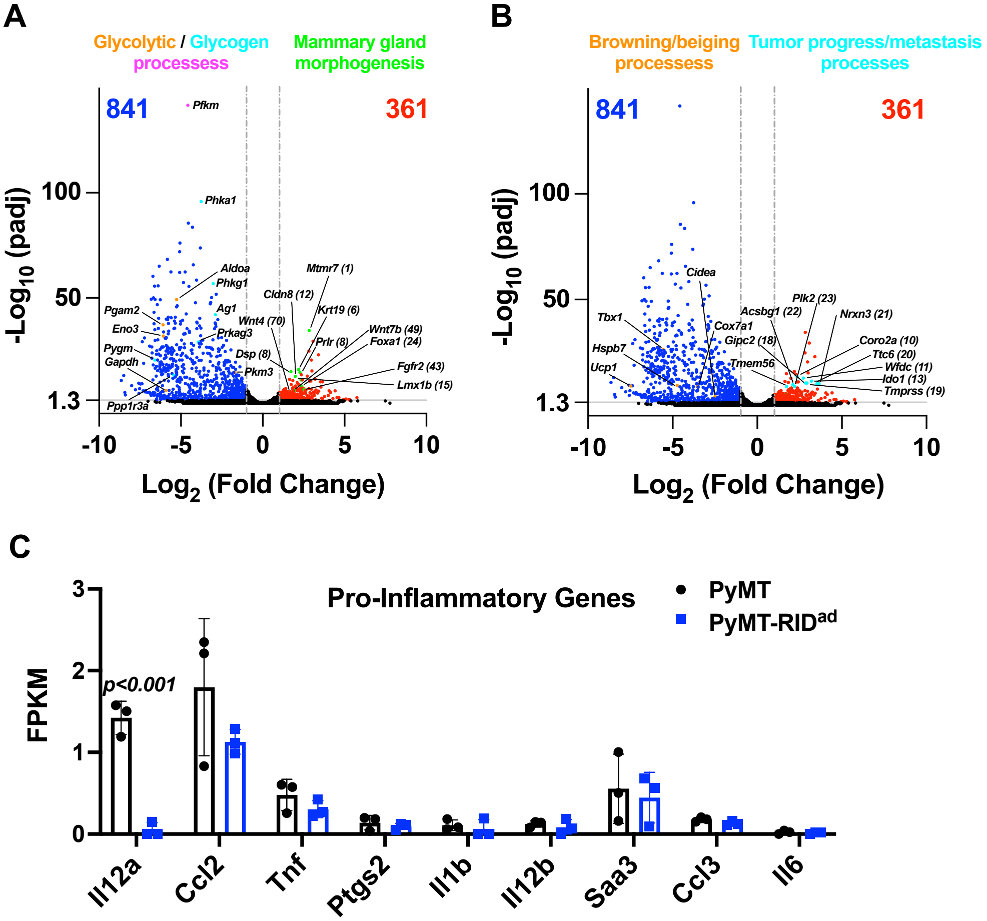

Supplemental Figure 19. Differentially expressed genes in the MFP of PyMT-RID[ad] mice compared to PyMT mice.

(A and B) Volcano plots comparing transcriptional profiles of the mammary fat pad (MFP) of PyMT-RID[ad] to PyMT mice. Related to Figure 7. Adjusted *p-value (padj)* ≤ *0.05* and absolute log2 fold change (|log2FoldChange|) ≥ 1.0 were applied in DESeq2 for differential gene expression analysis. While 361 genes were significantly upregulated, 841 genes were significantly downregulated. n = *3*/group. **(C)** RNA-seq analysis of inflammation-related mRNA expression in the MFP. n = *3*/group.

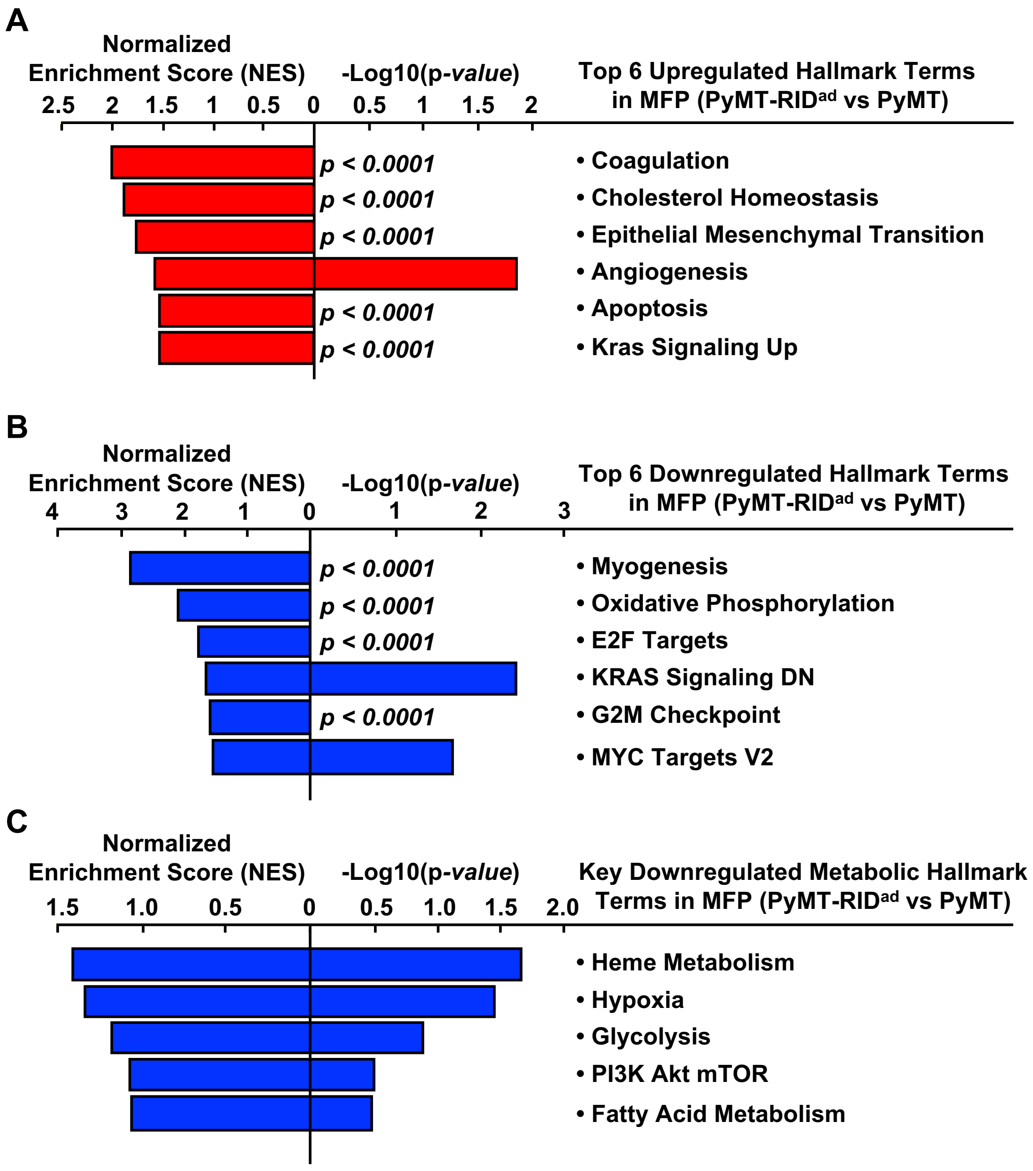

Supplemental Figure 20. Differentially expressed genes in the MFP of PyMT-RID^{ad} mice compared to PyMT mice.
(A) The top six positively enriched Hallmark terms in the mammary fat pad (MFP) of PyMT-RID^{ad} compared to PyMT mice. **(B)** The top six negatively enriched Hallmark pathways in the MFP of PyMT-RID^{ad} mice compared to PyMT mice. **(C)** The key downregulated Hallmark terms in the MFP of PyMT-RID^{ad} mice compared to PyMT mice. Normalized Enrichment Scores were calculated using the GSEA algorithm. Related to Figure 7. *n = 3*/group.

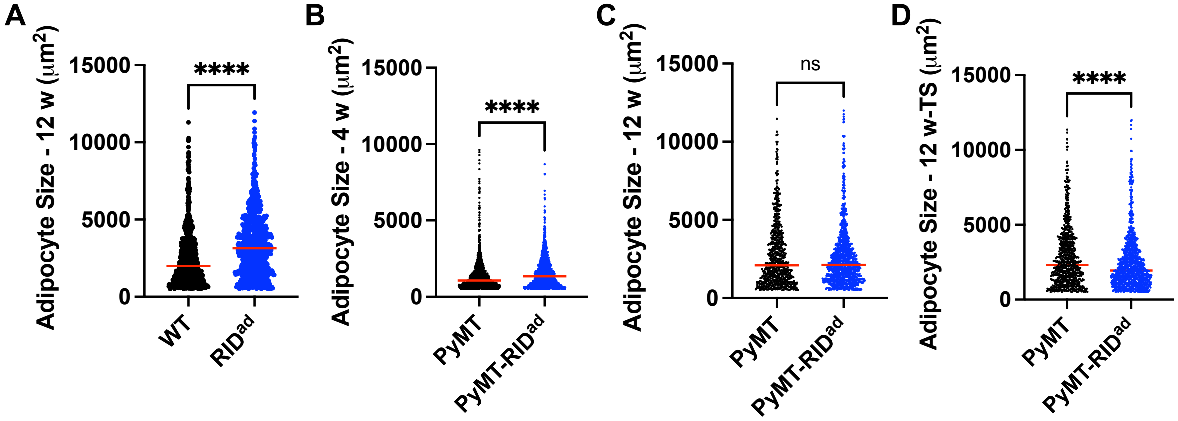

Supplemental Figure 21. Adipocyte size in RID^{ad} and PyMT-RID^{ad} mice.
(A-D) Adipocyte size in RID^{ad} and control mice after 12 weeks of Dox-HFD in the absence of PyMT (A), in PyMT-RID^{ad} and PyMT mice after 4 weeks of Dox-HFD (B), and in PyMT-RID^{ad} and PyMT mice after 12 weeks of Dox-HFD, assessed randomly (C) and near tumors (D). *n = 3-4*/group. TS: tumor surrounding. **(Statistics)** (A-D) Data are displayed as individual data points and mean and were analyzed by unpaired 2-tailed t-tests. ****$p<0.0001$.

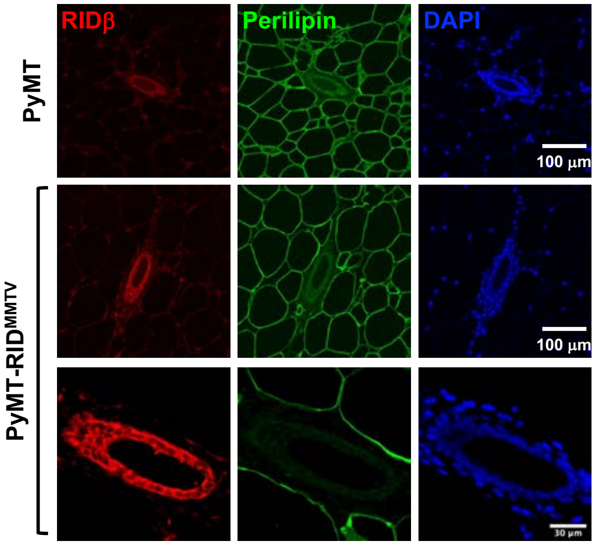

Supplemental Figure 22. RID expression in the MFP of RID^{MMTV} and PyMT mice.

Representative immunostaining of RIDβ and perilipin in the mammary fat pad (MFP) from the experiments shown in Figure 8B.

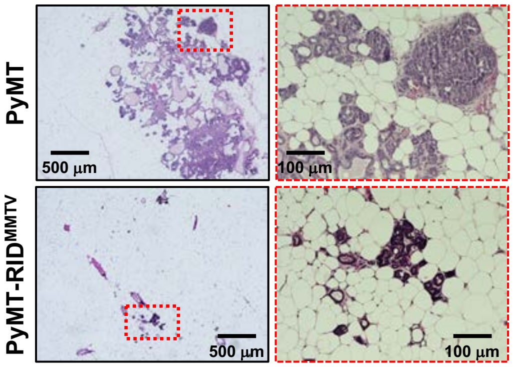

Supplemental Figure 23. Reduced abnormalities in mammary gland development in PyMT-RID^{MMTV} mice. H&E staining of the mammary fat pad (MFP).

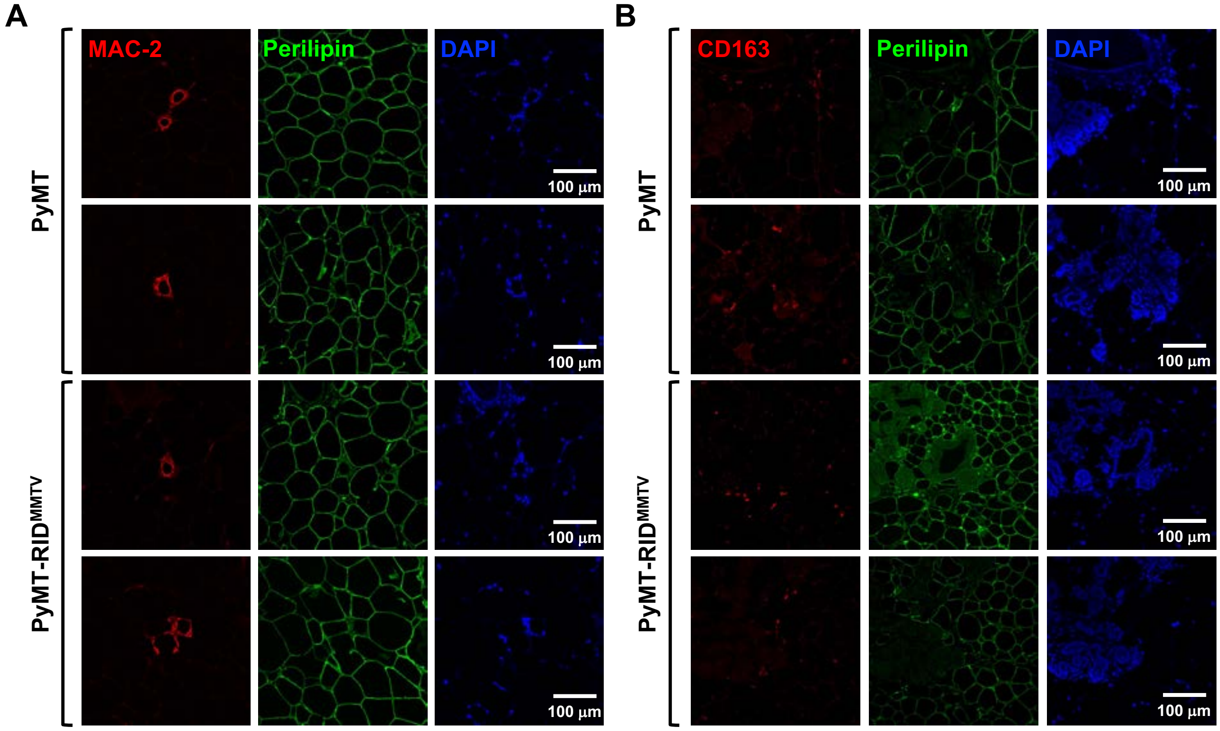

Supplemental Figure 24. Expression of MAC-2 and CD163 in the MFP of RID^{MMTV} and PyMT mice.

Representative immunostaining of MAC-2 and CD163 in the mammary fat pad (MFP) from the experiments shown in Figure 7H and J. *n* = *3*/group.

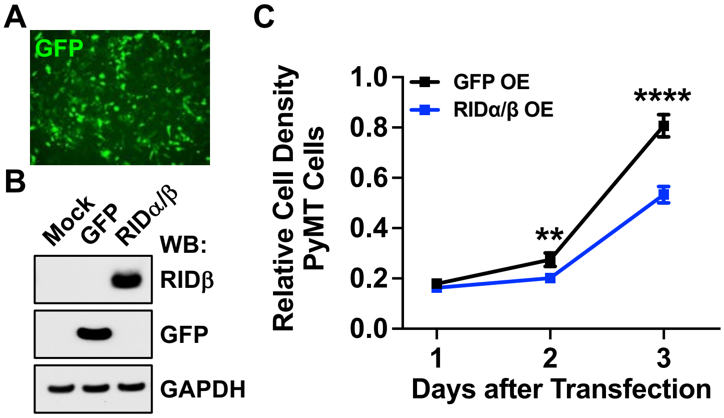

Supplemental Figure 25. RIDα/β overexpression suppresses the growth of immortalized tumor cells.
(A-C) Immortalized tumor cells were transfected with **RIDα/β or e**GFP (control) expression plasmids. (A) GFP expression 48 h after transfection **(B)** Western blot analysis of RID**β**, GFP, and GAPDH protein expression 48 h after transfection. **(C)** Proliferation assays. **(Statistics)** (C) Data are displayed as mean±SEM and were analyzed by two-Way ANOVA. ** *p<0.01, **** p<0.0001. n = 2*/group.

Supplemental Table 1. List of primers used for qPCR

Gene name	Forward primer	Reverse primer
Tnf	GAAAGGGGATTATGGCTCAGG	TCACTGTCCCAGCATCTTGTG
Il1b	GCAACTGTTCCTGAACTCAACT	ATCTTTTGGGGTCCGTCAACT
Ifng	TCAAGTGGCATAGATGTGGAAGAA	TGGCTCTGCAGGATTTTCATG
Nos2	GTTCTCAGCCCAACAATACAAGA	GTGGACGGGTCGATGTCAC
Il6	AAGCCAGAGTCCTTCAGAGAGA	ACTCCTTCTGTGACTCCAGCTT
Ccl2	AGCACCAGCCAACTCTCAC	TCTGGACCCATTCCTTCTTG
Mrc1	TGTGGTGAGCTGAAAGGTGA	CAGGTGTGGGCTCAGGTAGT
Mgl1	TGAGAAAGGCTTTAAGAACTGGG	GACCACCTGTAGTGATGTGGG
Arg1	CTCCAAGCCAAAGTCCTTAGAG	AGGAGCTGTCATTAGGGACATC
Adgre1	CTTTGGCTATGGGCTTCCAGTC	GCAAGGAGGACAGAGTTTATCGTG
36B4	AGATTCGGGATATGCTGTTGGC	TCGGGTCCTAGACCAGTGTTC
Rp16s	GATTTGCTGGTGTGGATATT	TCTTTGATCTCCTTCTTGGA

Supplemental Table 2. List of genotyping primer sequences

Mouse strain	Forward primer	Reverse primer
Adiponectin-rtTA	TGCAGGTCCTGATTGGATGTG	TTTCCTTGTCGTCAGGCCTTC
TRE-RID	CGGGACCGATCCAGCCTATC	TGCGCACACAAACCCAGTCA
MMTV-rtTA	GTTGGGGATTTAGCTCAGTG	GTACAGGGTAGGCTGCTCAA
MMTV-PyMT	GGAAGCAAGTACTTCACAAGGG	GGAAAGTCACTAGGAGCAGGG

Supplemental Table 3. Resources table

REAGENT or RESOURCE	SOURCE	IDENTIFIER
**ANTIBODIES**		
Cleaved Caspase-3	Cell Signaling	Cat #9661
RIDβ	Home-made	N/A
GPADH	Invitrogen	Cat #MA5-35235
β-Actin	Cell Signaling	Cat #4970
Perilipin	Fitzgerald	Cat #20R-PP004
Mac-2	BioLegend	Cat #125401
CD163	ProteinTech	Cat #16646-1-AP
Ki67	Abcam	Cat #ab15580
CD31	Invitrogen	Cat #14-0311-82
CD36	Invitrogen	Cat #PA1-16813
FABP4	Invitrogen	Cat #PA5-30591
ERα	Home-made	N/A
Alexa Fluor 488	Invitrogen	Cat #A-11006
Alexa Fluor 594	Invitrogen	Cat #A-11037
**CHEMICALS and OTHERS**		
Collagenase B	Roche	Cat #11088815001
Collagenase D	Roche	Cat #11088866001
DNase I	Zymo	Cat #E1011-A
Sulfosuccinimidyl Oleate	Cayman	Cat #1212012-37-7
PowerUp™ SYBR™ Green Master	Applied Biosystems	Cat #A25742
RPMI	Gibco	Cat #11875-093
DMEM/F12	Gibco	Cat #10565-018
Fetal bovine serum	GeminiBio	Cat #100-106
Normal goat serum	ThermoFisher	Cat #31873
Penicillin/streptomycin	Sigma-Aldrich	Cat #4458
RIPA Buffer	Pierce	Cat #89900
4-12% gradient polyacrylamide-SDS gel	Invitrogen	Cat #NP0336
Nitrocellulose membrane	BioRad	Cat #1704159
PrimeScript™ RT Master Mix	TaKaRa	Cat #RR036A
Antigen Unmasking Solution, Citrate-Based	Vector Labs	Cat #H-3300-250
VECTASHIELD mounting medium with DAPI	Vector Labs	Cat #H-2000
Hank's Balanced Salt Solution	Sigma-Aldrich	Cat #H8264
BSA	Sigma-Aldrich	Cat #A3294
Insulin	Eli Lilly	Cat #HI-210
Dextrose	Fischer Chemical	Cat #D16-1
3H-triolein	PerkinElmer	Cat #NET431001MC
GoTaq G2 Green Master Mix	Promega	Cat #M7823

Matrigel™ GFR Basement Membrane Matrix	Corning	Cat #CB-40230
TRIzol™ Reagent	Invitrogen	Cat #15596026
SuperSignal West Pico PLUS Chemiluminescent Substrate	ThermoFisher	Cat #34577
Formaldehyde Solution, 37%	ThermoFisher	Cat #S25329
8.0 um Transparent PET membrane	Corning	Cat #353097
Protease Inhibitor Cocktail	Millipore sigma	Cat #11873580001
Phosphatase inhibitor cocktail 3	Sigma	Cat #P0044
Formaldehyde Solution, 37%	Fishcerscientific	Cat #S25329
**CRITICAL COMMERCIAL ASSAYS**		
RNA purification kit	Qiagen	Cat #74104
EZ-10 DNAaway RNA miniprep kit	BIO BASIC	Cat #BS88136
Insulin ELISA	Crystal Chem	Cat #90080
Infinity Triglycerides Reagent	Thermo Scientific	Cat #703440
HR Series NEFA-HR(2)	FUJIFILM	Cat # 999-34691
**EXPERIMENTAL MODELS: CELL LINES**		
MCF-7	ATCC	HTB-22
MDA-MB-231	ATCC	HTB-26
HCC1954	ATCC	CRL-233
HCC38	ATCC	CRL-2314
EO771	Brekken Lab at the UTSW medical center	N/A
Met-1	Brekken Lab at the UTSW medical center	N/A
**EXPERIMENTAL MODELS: ORGANISMS/STRAINS**		
Adipoq-rtTA/*TRE-RID* mice	Zhu et al., 2020	N/A
Adipoq-rtTA/*TRE-RID*/*PyMT* mice	This study	N/A
MMTV-rtTA/*TRE-RID*/ *PyMT* mice	This study	N/A
**OLIGONUCLEOTIDES**		
Primers, see Supplemental Table 1and 2		
**Software and Algorithms**		
FIJI/ImageJ	NIH	RRID: SCR_003070
Prism10	GraphPad	RRID:SCR_002798
