# Supplementary material for: Inhibiting inflammation in adipocytes accelerates mammary tumor development in mice
Source: J Clin Invest. 2025 Jun 17;135(16):e187202. doi: 10.1172/JCI187202 (PMC12352902; doi:10.1172/JCI187202)

Full uncropped blots for Figure 1D

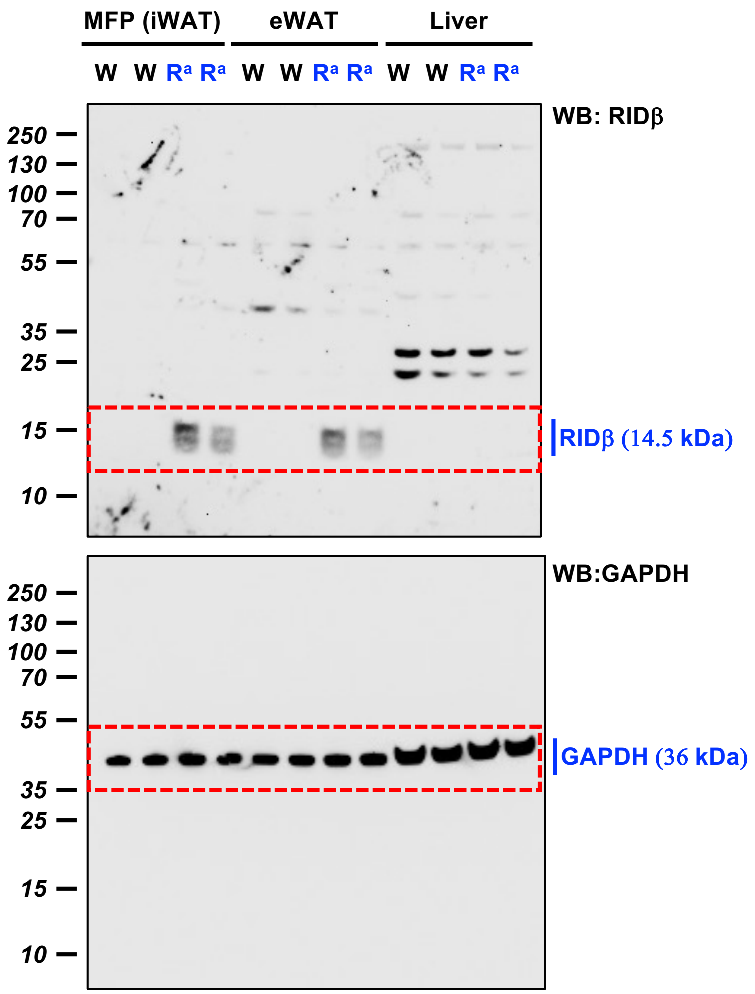

Full uncropped blots for Supplementray Figure 2A

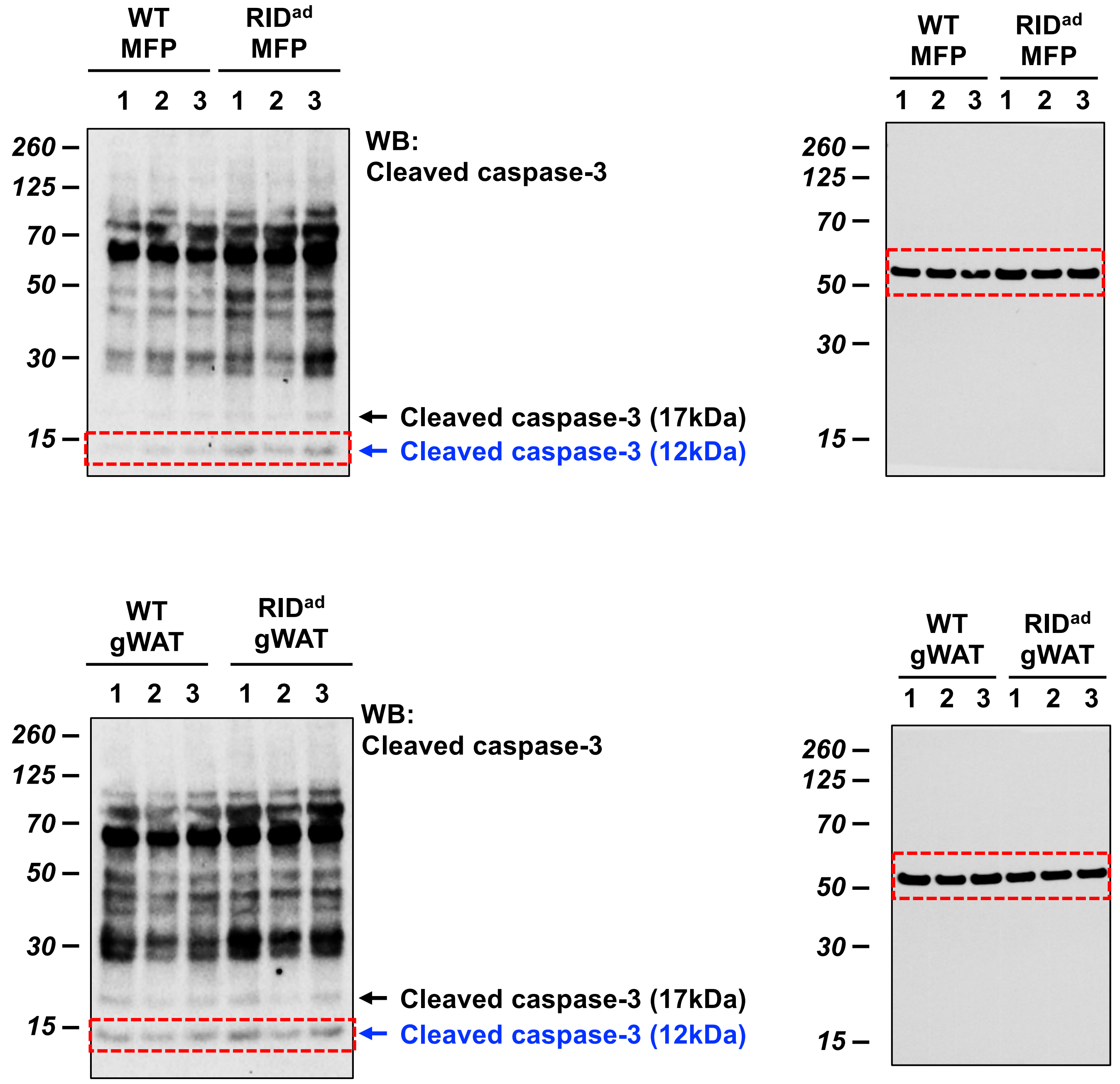

Full uncropped blots for Supplementary Figure 25B

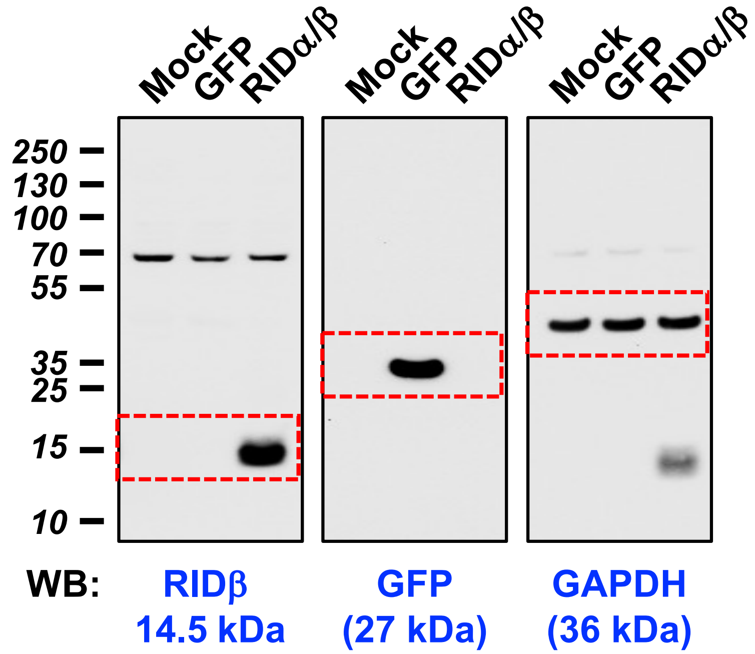

Supplement: Unedited blot and gel images [file jci-135-187202-s287.pdf]
